# Depression, violence and socioeconomic outcomes among refugees in East Africa: evidence from a multicountry representative survey

**DOI:** 10.1136/bmjment-2023-300773

**Published:** 2023-08-18

**Authors:** Julia R Pozuelo, Raphael Bradenbrink, Maria Flinder Stierna, Olivier Sterck

**Affiliations:** 1 Department of Global Health and Social Medicine, Harvard University, Cambridge, Massachusetts, USA; 2 Department of Psychiatry, University of Oxford, Oxford, UK; 3 MRC/Wits Rural Public Health and Health Transitions Research Unit (Agincourt), School of Public Health, Faculty of Health Sciences, University of the Witwatersrand, Johannesburg, South Africa; 4 Department of International Development, University of Oxford, Oxford, UK; 5 ODI (Overseas Development Institute), London, UK; 6 Institute of Development Policy, University of Antwerp, Antwerpen, Belgium

**Keywords:** Adult psychiatry, Depression & mood disorders

## Abstract

**Background:**

Existing research on refugee mental health is heavily skewed towards refugees in high-income countries, even though most refugees (83%) are hosted in low-income and middle-income countries. This problem is further compounded by the unrepresentativeness of samples, small sample sizes and low response rates.

**Objective:**

To present representative findings on the prevalence and correlates of depression among different refugee subgroups in East Africa.

**Methods:**

We conducted a multicountry representative survey of refugee and host populations in urban and camp contexts in Kenya, Uganda and Ethiopia (n=15 915). We compared the prevalence of depression between refugee and host populations and relied on regression analysis to explore the association between violence, depression and socioeconomic outcomes.

**Findings:**

We found a high prevalence of elevated depressive symptoms (31%, 95% CI 28% to 35%) and functional impairment (62%, 95% CI 58% to 66%) among the refugee population, which was significantly higher than that found in the host population (10% for depressive symptoms, 95% CI 8% to 13% and 25% for functional impairment, 95% CI 22% to 28%) (p<0·001). Further, we observed a dose–response relationship between exposure to violence and mental illness. Lastly, high depressive symptoms and functional impairment were associated with worse socioeconomic outcomes.

**Conclusion:**

Our results highlight that refugees in East-Africa—particularly those exposed to violence and extended exile periods—are disproportionately affected by depression, which may also hinder their socioeconomic integration.

**Clinical implications:**

Given the high prevalence of depression among refugees in East Africa, our results underline the need for scalable interventions that can promote refugees’ well-being.

**WHAT IS ALREADY KNOWN ON THIS TOPIC**

- Previous studies have found that refugees are more likely to experience depression, anxiety, and post-traumatic stress disorder than non-refugees. However, much of the existing evidence on the mental health of refugees comes from studies conducted in high-income countries, where only a minority of refugees live.
- Large and representative data on the prevalence and correlates of mental health among refugees in low-income and middle-income countries isare lacking.

**WHAT THIS STUDY ADDS**

- Our study aims to fill this gap by conducting a multi-country representative survey with refugees and host communities in urban and camp contexts in Kenya, Uganda, and Ethiopia.
- This study includes a representative and large sample, giving us enough statistical power to obtain precise estimates of this relationship that can be generalised to the other populations of interest.
- The survey contains a wealth of data on mental health, violence, and a range of socioeconomic outcomes, allowing us to provide insights into an overlooked relationship.

**HOW THIS STUDY MIGHT AFFECT RESEARCH, PRACTICE OR POLICY**

- Given the high prevalence of depression among refugees in East Africa and the limited access to mental health interventions, this study underscores the importance of early screening of refugees on arrival in exile and the vital need for expanding evidence-based prevention and treatment interventions to promote mental health and prevent a negative spiral of worsening outcomes over time.
- The findings in this study highlight the importance of studying depression, violence and socioeconomic outcomes jointly, a relationship that has so far remained relatively unaddressed among refugees in low-income and middle-income countries.
- Further, the results from this study suggest that increasing access to mental health interventions may create a virtuous cycle of increasing returns and provide an economic case for investing in refugees’ mental health.

## Background

At the end of 2021, 89.3 million people had been forced to flee their homes because of war, violence, fear of persecution and human rights violations.^1^ Refugees’ accumulation of traumatic and stressful experiences before, during and after fleeing may increase the likelihood of developing mental health disorders,^2^ which can persist years after displacement^3^ and hinder their socioeconomic integration.^4^ Measuring the prevalence of mental health issues in refugee populations and understanding its impacts on socioeconomic integration is important, especially in low-income settings, where the large majority of refugees live (83%) and where they often face additional adversities and limited access to treatment.^1 5^

To date, however, there is limited knowledge about the mental health of refugees in low-income and middle-income countries (LMICs). The existing research has three limitations. First, while 83% of the world’s refugees are hosted in LMIC, most studies focused on refugees living in high-income countries (HICs)^6^ (although there are important exceptions^7 8^). For example, it is particularly striking that 10 out of the 15 host countries considered in the recent review by Blackmore *et al* are HIC, and only 77 individuals (1.5%) out of the 5143 refugees considered in the meta-analysis are hosted in a low-income country (Uganda).^9^

Second, most studies are based on convenience samples, and therefore, do not provide a representative picture of mental health in the broader population of refugees.^10^ Small sample sizes and low response rates further compound this problem.^11^ Third, there is limited research on the association between depression and socioeconomic outcomes among refugees, especially in LMIC and camp contexts.^4^

To address these limitations, this study surveyed representative samples (n=8303) of the largest refugee populations in camps and cities of Kenya, Uganda and Ethiopia. Our sampling frame included Somali, Congolese and South-Sudanese refugees. We also surveyed representative samples of the host nationals in the nearby communities, which we used as a comparison group (n=7612).

### Objectives

This study had three main objectives. First, we explored the prevalence and correlates of depressive symptoms and functional impairment among different refugee subgroups and compared these to the prevalence found among the host population. Second, given that exposure to violence, such as torture, physical assault and armed conflict, is a well-established risk factor for poor mental health,^2^ we investigated the association between these types of violence exposure in the country of origin (predisplacement), depression and socioeconomic outcomes in exile (postdisplacement). Third, we examined the association between depression, violence exposure and socioeconomic outcomes, a relationship that has remained relatively unaddressed among refugees in LMIC.

## Methods

### Study design and population

**Figure 1:**
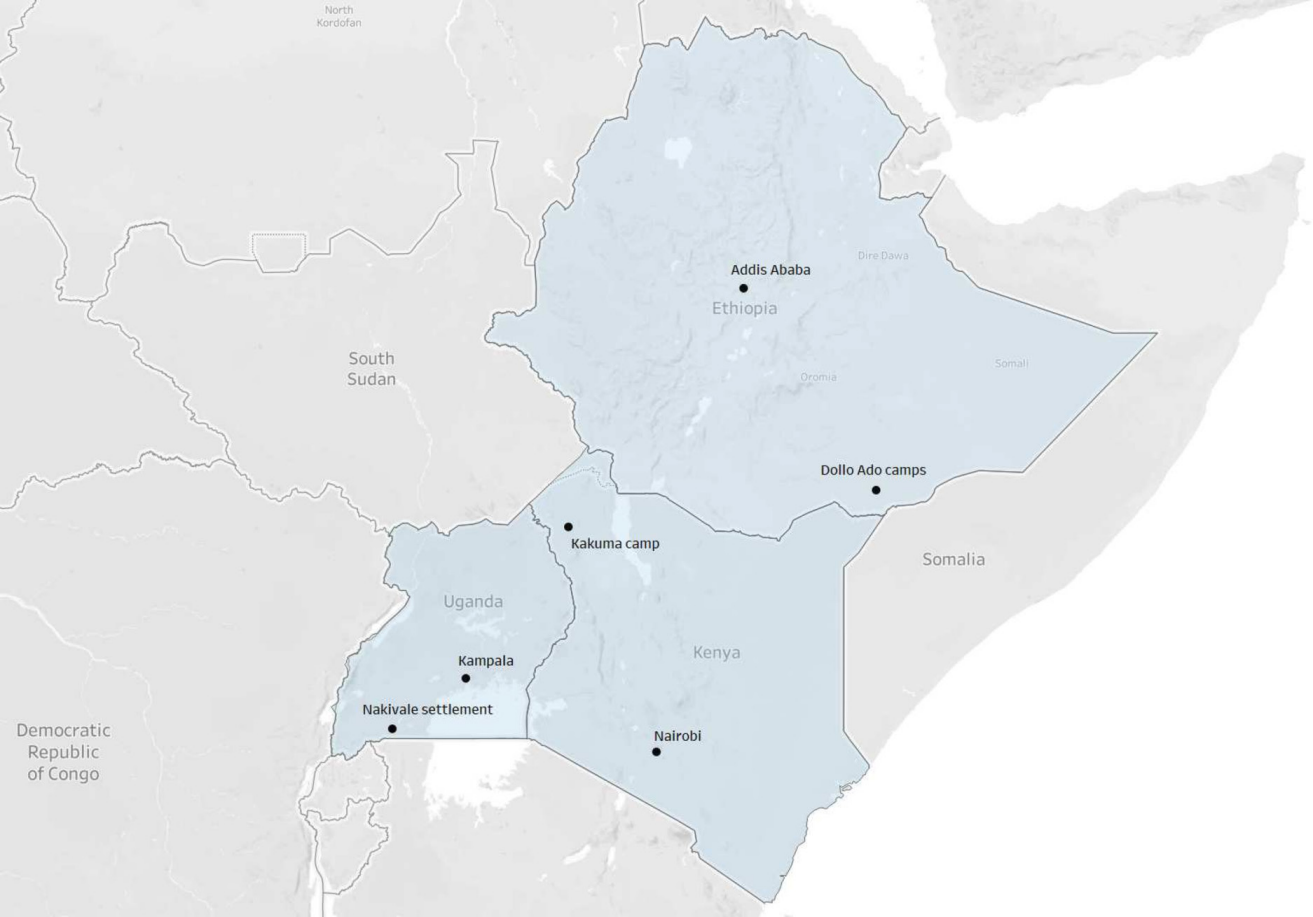
Survey locations in Ethiopia, Kenya and Uganda.

Between 2016 and 2018, we collected data on 15 915 refugees and members of the host populations living in Kenya, Uganda and Ethiopia.^12^ At the time of designing the research, in 2015, these three countries were hosting the largest number of refugees in Africa, hosting about 1.8 million refugees or 40% of all refugees in Africa.^13^ Our sampling frame covered the three capital cities—Nairobi, Kampala and Addis Ababa—and three groups of camps or settlements—the Kakuma camps in Kenya, the Nakivale settlement in Uganda and the Dollo Ado camps in Ethiopia (figure 1).

We surveyed representative samples of the largest refugee populations in each site using simple random or two-stage cluster sampling (n=8303). In all research sites in Kenya and Uganda, we surveyed Congolese and Somali refugees. In the Kakuma refugee camp, we also surveyed South-Sudanese refugees. In Ethiopia, we focused on Somali refugees in Addis Ababa and in the Dollo Ado camps. We also surveyed representative samples of the host nationals in the nearby communities, which we used as a comparison group (n=7612). Online supplemental table A.1 in the supplement describes the sampling methodology and sample sizes.

10.1136/bmjment-2023-300773.supp1 Supplementary data

### Measures

#### Depression

Following recent advances in clinical depression research, we considered two measures related to depression: symptoms and functioning.^14^

We used the Patient Health Questionnaire-9 (PHQ-9) to measure depressive symptoms.^15^ The PHQ-9 has been validated and widely used in various settings in African countries, including among refugees from Somalia, South Sudan and DR Congo.^16 17^ We consider and compare two measures in the analysis: the continuous score and a dichotomised score using the cut-off score of 10, indicating moderate to severe depressive symptoms. The internal consistency of the PHQ-9 was good, with a Cronbach’s α of 0.88 for the whole sample.

We used six questions from the WHO Disability Assessment Schedule 2.0 (WHODAS 2.0) to measure functional impairment in four domains of life (mobility, life activities, cognition and participation).^18^ WHODAS 2.0 has shown good reliability and validity and has been tested in various cultural settings.^18^ The internal consistency of the WHODAS 2.0 in our sample was high (0.82).

**Figure 2:**
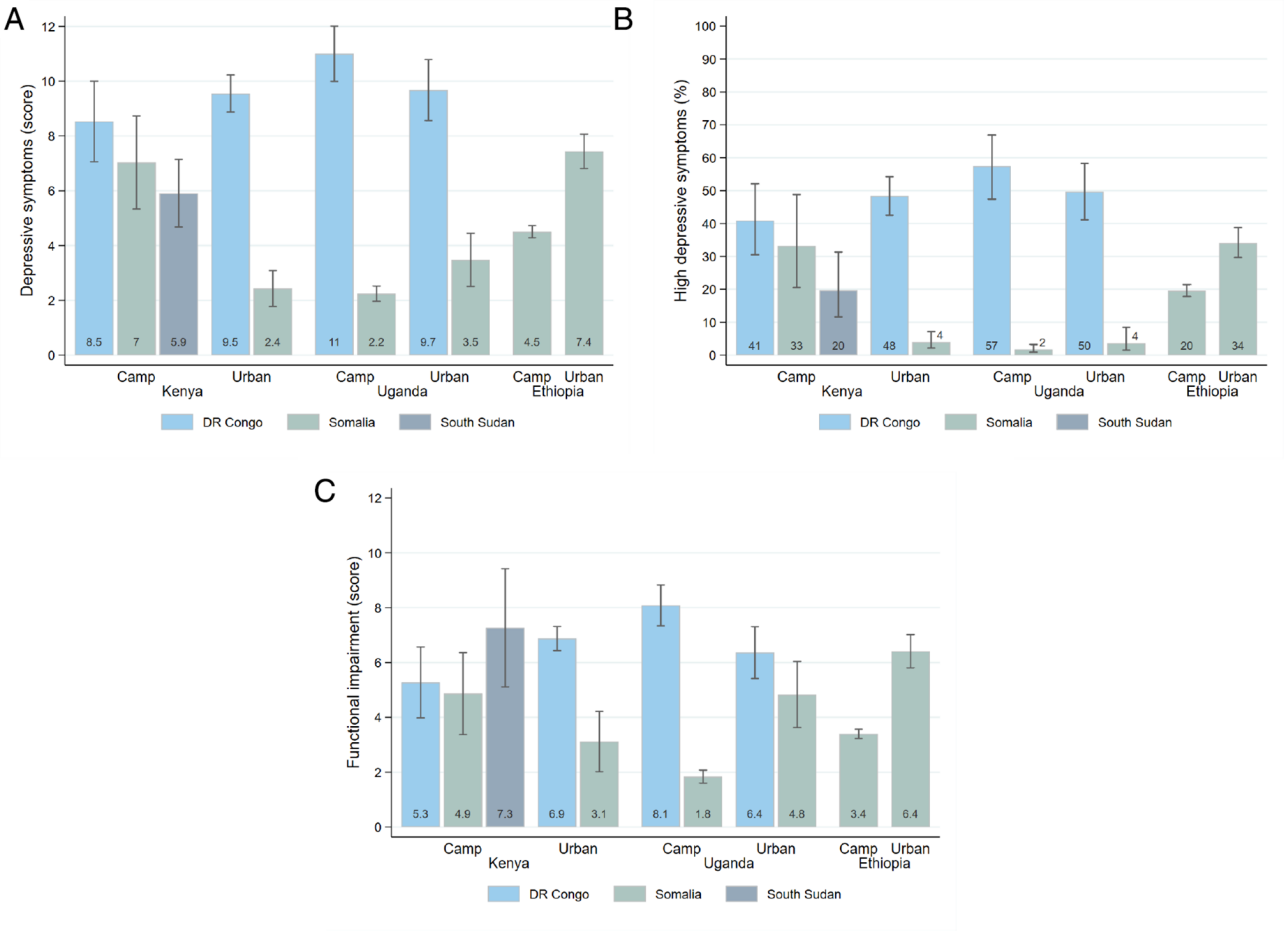
Measures of mental health and functioning. Note: (A) Shows the average PHQ-9 score for each refugee strata. (B) Shows the proportion of respondents from each refugee strata that have a PHQ-9 score of 10 or above, which is an indicator of moderate to severe depression. (C) Presents the average of functional impairment score, which aggregates respondents’ answers to six questions of the WHODAS 2.0 instrument. Data come from the Refugee Economies Dataset. PHQ-9, Patient Health Questionnaire-9; WHODAS 2.0, WHO Disability Assessment Schedule 2.0.

The distribution of these variables by nationality is shown in figure 2.

#### Exposure to violence

We relied on two measures of exposure to violence, one objective measure based on conflict event data and another subjective, self-reported measure of violent experiences.

The objective measure of refugees’ exposure to violence was constructed using the Armed Conflict Location and Event Data (ACLED).^19^ ACLED records incidents of political violence and protests reported by local, regional, or international news organisations, international organisations, NGOs, or trusted sources. We constructed an index capturing the number of all recorded violent events per 1000 inhabitants that occurred in refugees’ districts of origin in the 3 years preceding their exile.

The subjective measure of exposure to violence was constructed using self-reported information. Each respondent was asked whether they were ever (1) victims of violence, (2) victims of torture or (3) witnesses of violence or torture in their country of origin or in exile. We code respondents’ answers as 0 ‘no’ or 1 ‘yes’ and sum these to obtain a self-reported exposure to violence score ranging between 0 and 3. Cronbach’s α was 0.90 for this measure, indicating high reliability.

The distribution of the measures of exposure to violence is shown in online supplemental figures A.1 and A.2 in the supplement. There were significantly more violent events recorded in Somalia, and 62% of respondents self-reported some exposure to violence.

#### Socioeconomic outcomes

Recognising that socioeconomic well-being is a multifaceted concept that is complex to measure, our analysis focused on four outcomes that capture different life aspects. First, we considered employment status as a binary variable identifying respondents who reported having an income-generating activity (IGA) (whether employment or self-employment). Second, we used a measure of income from work, expressed in constant 2015 USD, and winsorised at the 99th percentile to limit the influence of outliers. When analysing this variable, we considered the entire sample and a restricted sample of refugees with an IGA. With the entire sample, the estimated relationship combines the ‘effects’ on employment status and work income; the restricted sample allows us to study the ‘effects’ on work income conditionally on having an IGA. Third, we considered a measure of life satisfaction, constructed using answers to the following question: ‘All things considered, how satisfied are you with your life as a whole these days?’. Possible answers range from 1, ‘very unsatisfied’, to 5, ‘very satisfied’. Finally, we used the Individual Dietary Diversity Score (IDDS) to measure the variety of respondents’ food intake.^20^ The IDDS is calculated by counting the number of twelve types of food consumed at any time within the 7 days preceding the survey, resulting in a score ranging from 0 to 12.

#### Covariates

We included a long series of control variables in regression analysis to minimise the risk of omitted variable bias. These variables include individual-level and household-level characteristics (eg, age, sex, marital status, education, years in exile and household size).

See online supplemental tables A.2-A.4 in the supplement for a detailed description of all variables considered in the analysis and descriptive statistics.

### Statistical analysis

The first part of the analysis explored the prevalence of depressive symptoms and functional impairment in refugee populations. We used simple t-tests to compare the prevalence in refugee subgroups and refugee and host populations. We report 95% CIs.

The second part of the analysis used regression analysis to explore the association between exposure to violence predisplacement and mental health and socioeconomic outcomes postdisplacement. We estimated regression equations of the following form:

$$Y_{i}=\alpha +\beta \;V_{i}+\delta^{T}\;X_{i}+EA_{j}+EN_{k}+\varepsilon_{i}$$

where $Y_{i}$ is the dependent variable (either a measure of depressive symptoms, functional impairment or socioeconomic outcomes), and $V_{i}$ is the explanatory variable of interest (either a measure of violence exposure, depressive symptoms or functional impairment). $X_{i}$ is a vector of control variables, which are described in online supplemental tables A.2-A.4 in the supplement. ${EA}_{j}$ and ${EN}_{k}$ are enumeration areas and enumerator fixed effects, respectively. We included control variables and fixed effects to minimise the risk of omitted variable bias. Enumeration area fixed effects were included to control for unobserved variables that could influence the average answers obtained in the different locations. Enumerator fixed effects are useful to control for unobserved variables that could influence each enumerator’s average answers.

We were primarily interested in the parameter β , estimated using ordinary least squares (OLS) regression. Given the absence of random or quasi-random variation in violence exposure, depressive symptoms and functional impairment, omitted variable bias and reverse causality are possible. We were, therefore, cautious when interpreting regression results and avoided making strong causal claims.

We used sampling weights and clustered standard errors (SEs) in the analysis to account for the sampling design. Variables that are non-binary were standardised to facilitate the interpretation and comparison of regression coefficients.

This analysis adheres to the Strengthening the Reporting of Observational Studies in Epidemiology (STROBE) guideline. The STROBE checklist is shown in online supplemental file.

## Findings

We observed a higher prevalence of elevated depressive symptoms and functional impairment among refugees than in the general population. On average, 31% of refugees were moderately or severely depressed (PHQ-9 score ≥10) (95% CI 28% to 35%), compared with 10% in the host population in the same contexts (95% CI 8% to 13%). Significant differences existed between contexts and nationalities, as shown in figure 2. Being female, older and the duration of exile were associated with higher PHQ-9 scores in regression analysis (online supplemental table A.9 in the supplement).

Controlling for survey location, refugees scored 0.57 SD higher than host populations on the functional impairment score (95% CI 0.42 to 0.71, p<0.001). A significant proportion of refugees reported moderate to severe difficulties in walking (35%, 95% CI 29% to 40%), standing (21%, 95% CI 18% to 24%), taking care of household responsibilities (31%, 95% CI 27% to 35%), concentrating (22%, 95% CI 18% to 26%), learning (27%, 95% CI 23% to 31%) and joining community activities (24%, 95% CI 21% to 28%). Similar to depressive symptoms, we also observed significant differences across nationalities and sites for functional impairment (figure 2). The degree of functional impairment was significantly higher for females, older people and those with extended exile periods (online supplemental table A.11 in the supplement).

A large majority of respondents self-reported having been exposed to violence or torture (72% CI 68% to 76%), either as a direct victim (62% CI 58% to 65%) or witness (64% CI 59% to 68%). We observe important differences between contexts and nationalities (online supplemental figures A.1 and A.2 in the supplement). While being older was associated with a higher degree of self-reported exposure to violence, we found no significant association between sex and self-reported exposure to violence.

**Figure 3:**
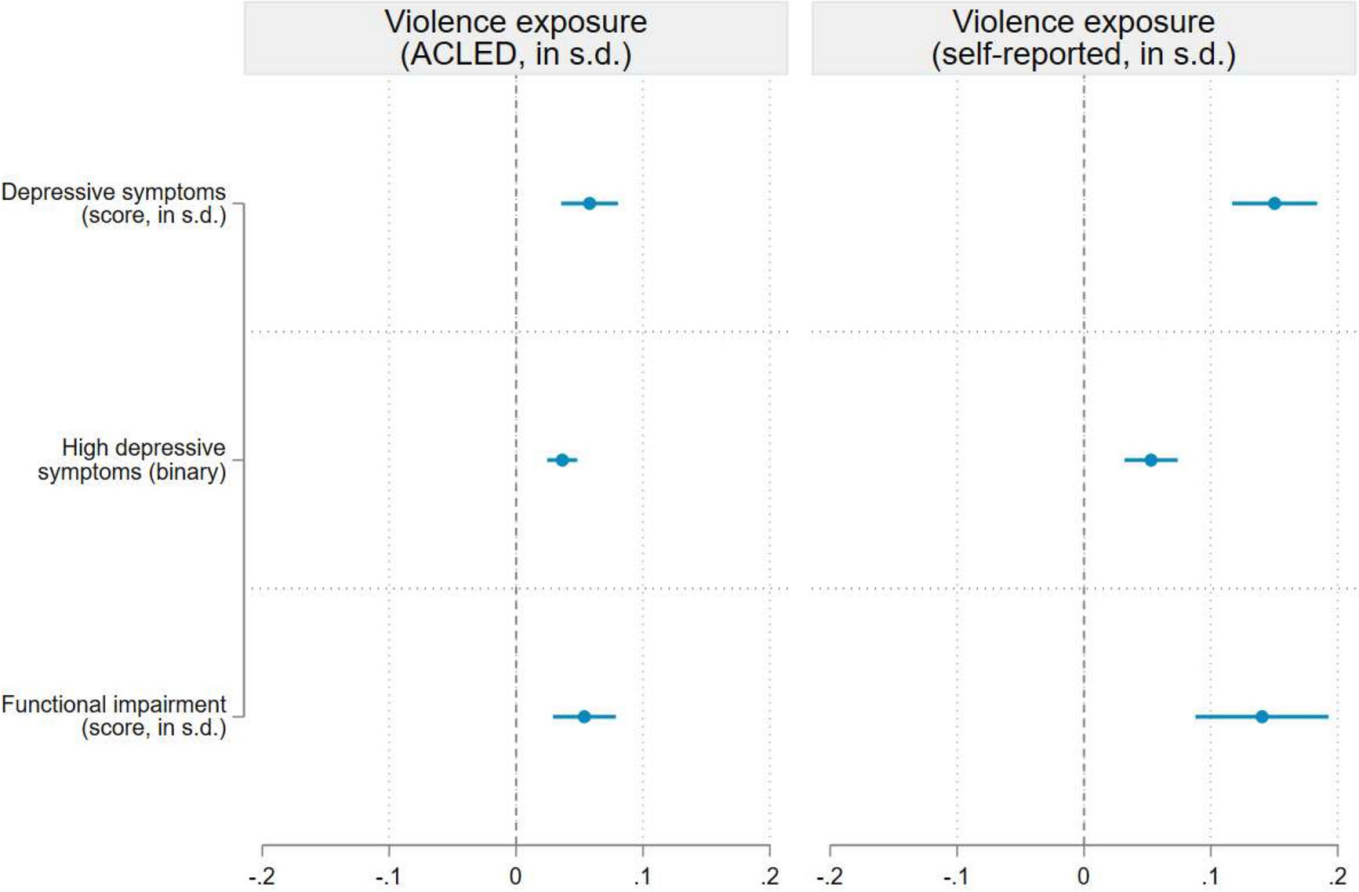
Association between exposure to violence and depressive symptoms. Note: This figure presents the results of the ordinary least squares (OLS) regressions shown in columns (2) and (4) of online supplemental table A.6 in the supplement. Three outcome variables are analysed. Depressive symptoms are measured by the PHQ-9 score (standardised score). High depressive symptoms are measured by a binary variable equal to 1 if the PHQ-9 score ≥10, and 0 otherwise (binary variable). The functional impairment score aggregates six questions from the WHODAS 2.0 (standardised score). Two explanatory variables are considered: exposure to violence based on ACLED data (standardised score) and self-reported exposure to violence (standardised score). Regressions include control variables (described in online supplemental table A.3 in the supplement), enumerator fixed effects, and enumeration area fixed effects. Sampling weights are included in all regressions and 95% cluster-robust CIs are reported. ACLED, Armed Conflict Location and Event Data; PHQ-9, Patient Health Questionnaire-9; WHODAS 2.0, WHO Disability Assessment Schedule 2.0.

Regression analysis showed that respondents who faced higher levels of violence were more likely to experience high depressive symptoms in exile (figure 3). All regression coefficients were positive and statistically significant at the 1% level, showing that violence exposure was associated with poor mental health. With the self-reported measure of violence, we find that a 1 SD increase in experience of violence was associated with a 0.15 SD increase in the PHQ-9 score (95% CI 0.12 to 0.18, p<0.001) and a 0.14 SD increase in the functional impairment score (95% CI 0.09 to 0.19, p<0.001). The association between self-reported exposure to violence and the PHQ-9 score is largely driven by direct exposure to violence or torture rather than witnessing violence or torture (online supplemental table A.25 in the supplement). Effect sizes are substantially smaller when using the objective measure of violence exposure based on ACLED. This is most likely due to attenuation bias because the ACLED-based index captures violence at the district level and hence does not measure whether respondents personally experienced violence.^21 22^

**Figure 4:**
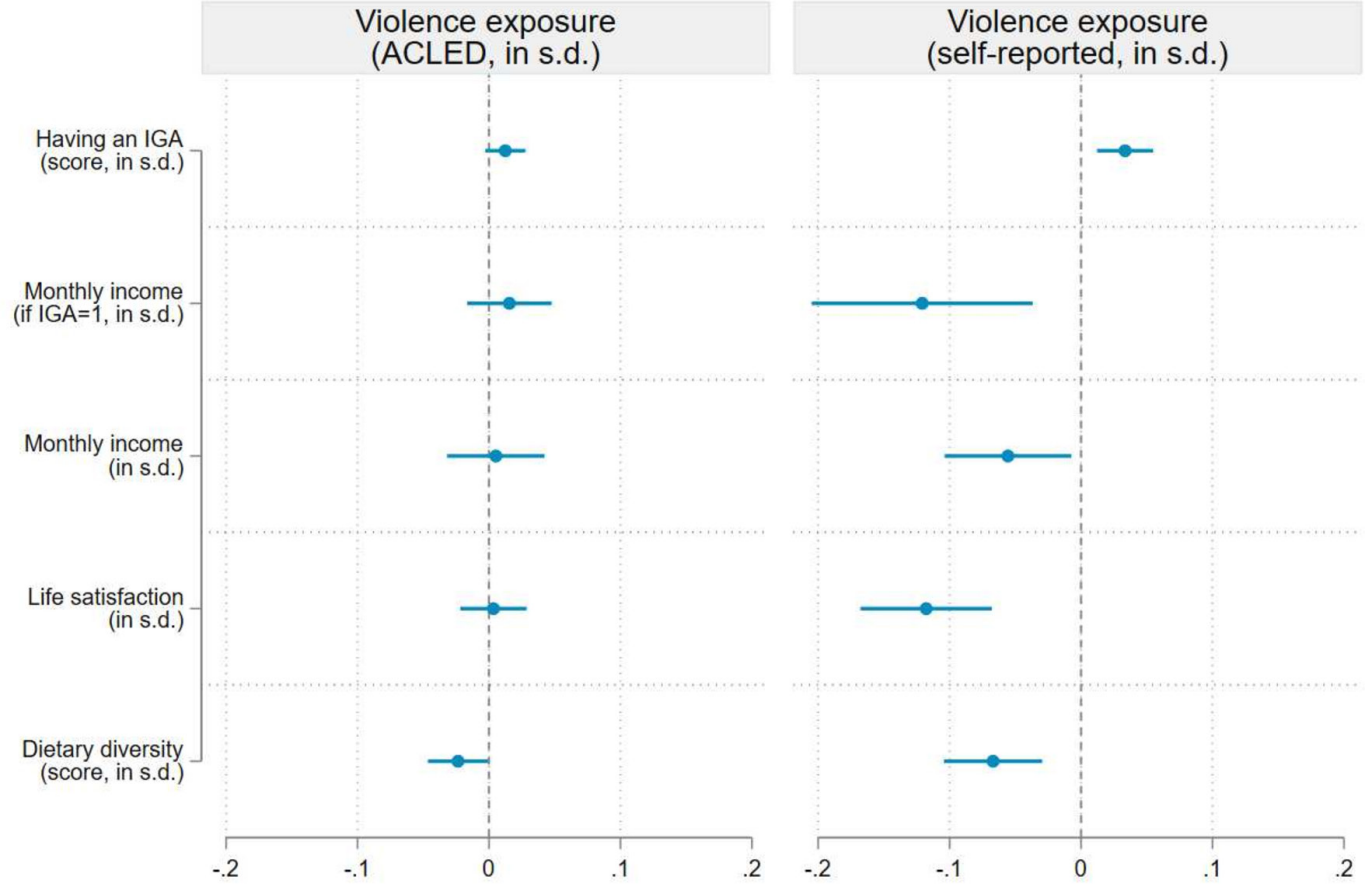
Association between exposure to violence and socioeconomic outcome. Note: This figure presents the results of the ordinary least squares (OLS) regressions shown in columns (2) and (4) of online supplemental table A.7 in the supplement. Five outcome variables are analysed: a dummy variable equal to 1 if the respondent has a income generating activity (IGA), work income conditional on having an IGA, work income (unconditional), life satisfaction and the individual dietary diversity score (all outcomes are standardised). Two explanatory variables are considered: exposure to violence based on ACLED data (standardised score) and self-reported exposure to violence (standardised score). Regressions include control variables (described in online supplemental table A.3 in the supplement), enumerator fixed effects, and enumeration area fixed effects. Sampling weights are included in all regressions and 95% cluster-robust confidence intervalCIs are reported. ACLED, Armed Conflict Location and Event Data.

Violence exposure was also a significant predictor of socioeconomic outcomes in our sample, but only when the self-reported measure of violence exposure was considered (figure 4). Again, the ACLED-based measure might be too indirect to adequately capture respondents’ experience of violence before exile, leading to attenuation bias. We find that a 1 SD increase in the self-reported measure of exposure to violence is associated with an increase of 3.3 percentage points in the likelihood of having an IGA (95% CI 1.2 to 5.5, p=0.002), but a 0.12 SD reduction in work income conditionally on having a job (95% CI −0.20 to −0.04, p=0.005). The combination of these two opposite effects on unconditional income is negative and statistically significant at the 5% level: a 1 SD increase in the self-reported measure of exposure to violence is associated with a 0.06 SD reduction in income (95% CI −0.10 to −0.007, p=0.02). We provide a possible explanation for these associations in the discussion section. We also find that a 1 SD increase in the self-reported measure of violence is associated with a 0.12 SD reduction in life satisfaction (95% CI −0.17 to −0.07, p<0.001) and a 0.07 SD reduction in dietary diversity (95% CI −0.10 to −0.03, p<0.001). While statistically significant at conventional levels, the magnitude of these associations is relatively small. The associations between self-reported exposure to violence and income, life satisfaction and dietary diversity are also largely driven by direct exposure to violence or torture rather than witnessing violence or torture (online supplemental table A.26 in the supplement).

**Figure 5:**
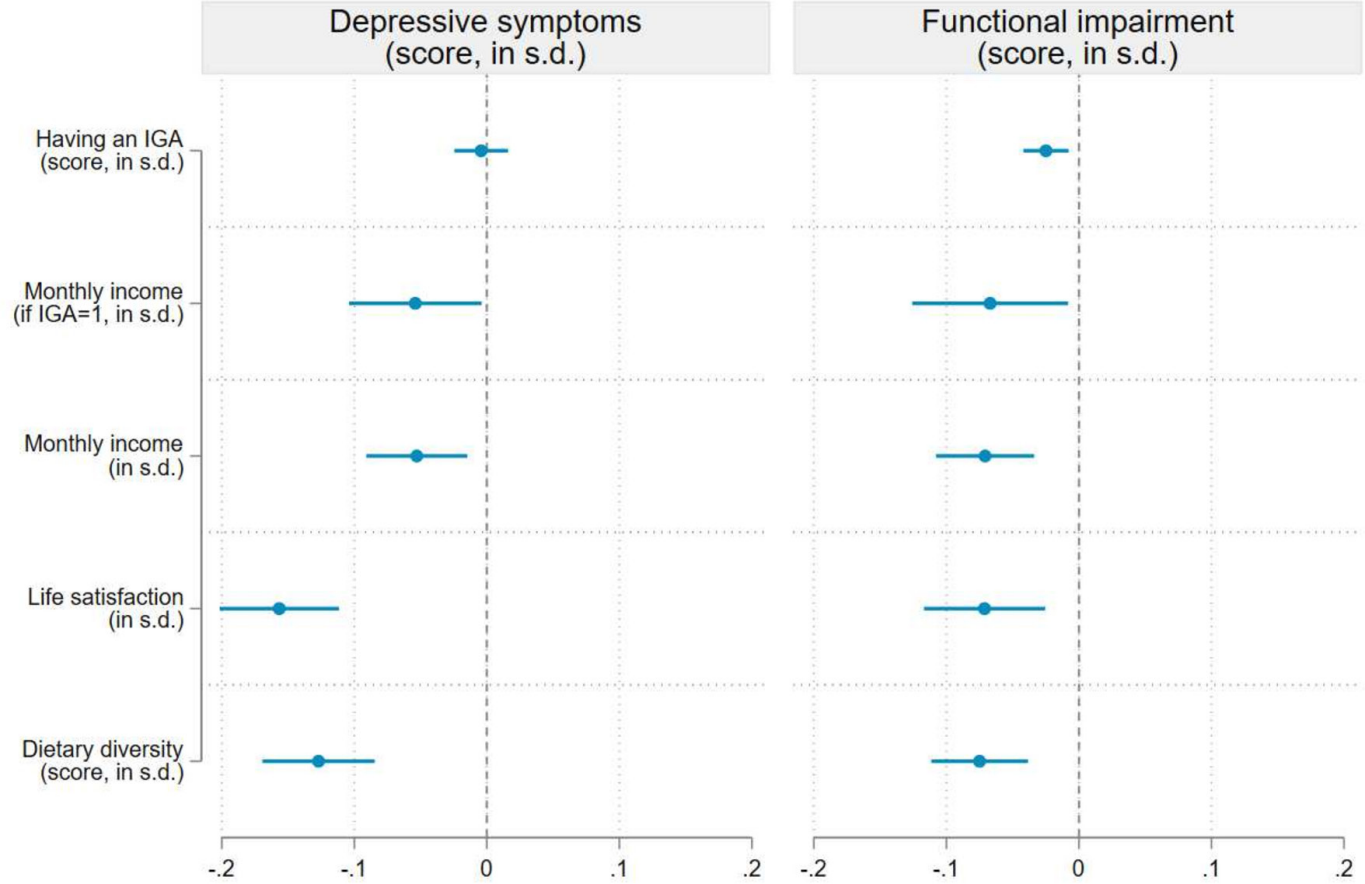
Association between exposure to depressive symptoms and socioeconomic outcomes. Note: This figure presents the results of the ordinary least squares (OLS) regressions shown in columns (2) and (6) of online supplemental table A.8 in the supplement. Five outcome variables are analysed: a dummy variable equal to 1 if the respondent has a income generating activity (IGA), work income conditional on having an IGA, work income (unconditional), life satisfaction and the individual dietary diversity score (all outcomes are standardised). Two explanatory variables are considered: depressive symptoms measured by the PHQ-9 score (standardised score) and a functional impairment score based on the WHODAS 2.0 (standardised score). Regressions include control variables (described in online supplemental table A.3 in the supplement), enumerator fixed effects, and enumeration area fixed effects. Sampling weights are included in all regressions and 95% cluster-robust CIs are reported. PHQ-9, Patient Health Questionnaire-9; WHODAS 2.0, WHO Disability Assessment Schedule 2.0.

Depressive symptoms and functional impairment were also significant predictors of socioeconomic outcomes in our sample (figure 5). High PHQ-9 scores were associated with lower incomes, life satisfaction and dietary diversity. We find that a 1 SD increase in the PHQ-9 score is associated with a 0.05 SD reduction in income (95% CI −0.09 to −0.01, p=0.07), a 0.16 SD reduction in life satisfaction (95% CI −0.20 to −0.11, p<0.001) and a 0.13 SD reduction in dietary diversity (95% CI −0.17 to −0.07, p<0.001). Functional impairment was also associated with a lower likelihood of having an IGA, lower incomes, lower life satisfaction and lower dietary diversity. These correlations should be interpreted prudently as reverse causation is possible, that is, socioeconomic outcomes can influence mental health.

## Discussion

Pre-existing evidence on refugee mental health is heavily skewed towards refugees living in HIC, even though 83% of the world’s refugees live in LMIC. Our study addressed this research gap by conducting a multicountry survey of refugees (N=8303) and the host population (N=7612) to present representative findings on the prevalence and correlates of depression among refugees in East Africa.

This research article contributes significantly to the existing literature through several key advancements. First, it addresses a critical gap by focusing on refugees in East Africa, a population that has been understudied and marginalised despite its substantial size. Second, it covers refugees and host communities in urban and camp contexts within three major host countries, filling a significant gap in the literature. Third, this study includes a representative and large sample, giving us enough statistical power to obtain precise estimates of this relationship that can be generalised to the other populations of interest. Finally, the research instruments used in the survey encompassed a wide range of measures, enabling in-depth exploration of the relationships between depression, violence exposure and socioeconomic outcomes. These methodological advancements enhance the rigour and comprehensiveness of the study, ultimately contributing to a deeper understanding of the mental health challenges faced by refugees in East Africa.

We highlight three novel findings. First, we found a high prevalence of depressive symptoms (31%, 95% CI 28% to 35%) and functional impairment (62%, 95% CI 58% to 66%) among refugees, which is comparable with rates measured for refugees hosted in high-income countries.^2 9^ The observed prevalence was significantly higher than that found among the host population living in the same contexts (10% for depression, 95% CI 8% to 13%; and 25% for functional impairment, 95% CI 22% to 28%). Second, our analysis establishes a dose–response relationship between exposure to violence and mental illness in LMIC, whereby the severity of mental illness increases as refugees’ exposure to traumatic experiences increases.^23^ More than half of the sample reported having been exposed to violence, which was a significant predictor of mental illness and socioeconomic outcomes during exile. We found that violence exposure was associated with an increased probability of having a job, although their income was comparatively lower than those who did not experience violence. One possible explanation for these associations is that refugees who experienced violence might be more aware of the limited chances of returning to their country of origin and may invest more resources into finding employment, even if the pay is low. Consistent with this explanation, we find that self-reported violence exposure is negatively associated with the expectation to live in the country of origin again in the next 3 years (p=0.04). Third, higher depressive symptoms and functional impairment were associated with worse socioeconomic outcomes, including higher unemployment, lower wages, lower life satisfaction and a less diverse diet, which aligns with previous studies conducted among the general population.^24^

We note several limitations of the study. First, this study explores the association between mental health, violence and socioeconomic outcomes using observational evidence, and thus, we cannot infer causality. While we included a long list of control variables and fixed effects in the analysis, our results could be affected by omitted variables bias and reverse causation. Second, as this is a cross-sectional study, we could not determine the temporal relationship between mental health and socioeconomic outcomes. However, we relied on conflict event data from ACLED to establish temporal precedence between exposure to violence before fleeing and all other outcomes. Third, our study focused on depressive symptoms and functional impairment, leaving clinical diagnosis of depression and other mental health issues for future research (eg, post-traumatic stress disorder and anxiety disorders). It also explored one cause of displacement (conflict related), ignoring other causes, such as climate-related events.

## Clinical implications

Our results highlight that refugees—particularly those exposed to violence—are disproportionately affected by depression, one of the most disabling and costly illnesses worldwide.^25^ They are also more likely to have worse socioeconomic outcomes. While the effect sizes of the associations between exposure to violence, mental health and socioeconomic outcomes were relatively small in magnitude (≤0.2 SD), the ongoing impact of mental illness over time can be significant. Further, mental health issues appear to persist and accumulate with a lengthier duration of exile, indicating that postmigration conditions matter and that interventions that address broader socioeconomic conditions of refugees may be particularly effective.^26^ Our results also underscore the importance of early screening for mental illness and the vital need for expanding evidence-based prevention and treatment interventions to promote mental health and prevent a negative spiral of worsening outcomes over time.^27^ Given the high comorbidity of depression with other psychiatric disorders among refugees,^6^ transdiagnostic approaches may also provide a promising opportunity to address multiple mental health symptoms (rather than focusing explicitly on one disorder).^28 29^

Based on our results, we hypothesise that treatment of mental illness among refugees may be effective at improving both mental health and socioeconomic outcomes. This has been supported by recent reviews that demonstrate that mental health interventions were not only less expensive than many economic interventions but also had similar (or even larger) effects on mental health and socioeconomic outcomes.^30^ These findings, combined with the results from this study, suggest that developing high-quality mental health interventions for refugees may create a virtuous cycle of increasing returns and provide an economic case for investing in mental health.^25^

## Data Availability

Data are available in a public, open access repository. Deidentified data and replication files will be made available on the authors’ personal websites after the publication of the study.
