# Supplementary material for: Depression, violence and socioeconomic outcomes among refugees in East Africa: evidence from a multicountry representative survey
Source: BMJ Ment Health. 2023 Aug 18;26(1):e300773. doi: 10.1136/bmjment-2023-300773 (PMC10577724; doi:10.1136/bmjment-2023-300773)
Supplement: Supplementary data [file bmjment-2023-300773supp001.pdf]

Supplement - Depression, Violence, and Socioeconomic Outcomes among Refugees in East Africa: Evidence from a Multi-Country Representative Survey

Table	Page
Table A.1 – Refugee Sampling Strategies	2
Table A.2 – Construction of variables	3
Table A.3 – Descriptive Statistics (Refugee Sample)	4
Table A.4 – Descriptive Statistics by Nationality (Refugee Sample)	5
Table A.5 – Comparison of Different Measures of Violence Exposure	6
Table A.6 – Association between exposure to violence and depression, with standardized variables	7
Table A.7 – Association between exposure to violence and socioeconomic outcomes, with standardized variables	8
Table A.8 – Association between depression and socioeconomic outcomes, with standardized variables	9
Table A.9 – Association between exposure to violence and the depressive symptoms score, with standardized variables	10
Table A.10 – Association between exposure to violence and the prevalence of high depressive symptoms, with standardized variables	11
Table A.11 – Association between exposure to violence and functional impairment, with standardized variables	12
Table A.12 – Association between exposure to violence and having an income-generating activity (IGA), with standardized variables	13
Table A.13 – Association between exposure to violence and income conditional on having an IGA, with standardized variables	14
Table A.14 – Association between exposure to violence and income, with standardized variables	15
Table A.15 – Association between exposure to violence and life satisfaction, with standardized variables	16
Table A.16 – Association between exposure to violence and dietary diversity, with standardized variables	17
Table A.17 – Association between mental health and having an IGA, with standardized variables	18
Table A.18 – Association between mental health and income conditional on having an IGA, with standardized variables	19
Table A.19 – Association between mental health and income, with standardized variables	20
Table A.20 – Association between mental health and life satisfaction, with standardized variables	21
Table A.21 – Association between mental health and dietary diversity, with standardized variables	22
Table A.22 – Association between exposure to violence and depression	23
Table A.23 – Association between exposure to violence and socioeconomic outcomes	24
Table A.24 – Association between depression and socioeconomic outcomes	25
Table A.25 – Association between self-reported exposure to violence and depression, with standardized variables	26
Table A.26 – Association between self-reported exposure to violence and socioeconomic outcomes, with standardized variables	27
Figure A.1 Exposure to violence	28
Figure A. 2 Components of self-reported exposure to violence	29
STROBE Statement	30

Pozuelo JR, *et al. BMJ Ment Health* 2023; 26:e300773. doi: 10.1136/bmjment-2023-300773

Table A.1 – Refugee Sampling Strategies

Sub-population	Sampling strategy	Refugee sub-population (UNHCR data)	Sample size Individuals (HHs)
**Kenya Kakuma - Total (November-December 2016)**			**1,362 (480)**
Somali refugees	Household sampling: We used two-stage cluster sampling using UNHCR registration data. We first randomly selected 20 blocks in the camp (sampling proportional to size with replacement). Following a mapping exercise of each selected block, we randomly selected 8 households on each map.<br>Within-household sampling: In households with less than 5 adults, all adults were interviewed. When the number of adults was higher than 5, we interviewed the household head as well as 4 other adults randomly selected.	40,074 individuals (26% of camp)	456 (160)
South-Sudanese refugees	Household sampling: Similar to the sampling strategy used for Somali refugees in Kakuma.<br>Within-household sampling: Same procedure as for Somali refugees in Kakuma.	82,339 individuals (54% of camp)	463 (160)
Congolese refugees	Household sampling: Similar to the sampling strategy used for Somali refugees in Kakuma.<br>Within-household sampling: Same procedure as for Somali refugees in Kakuma.	9,171 individuals (6% of camp)	443 (160)
**Kenya Nairobi - Total (May 2017)**			**1,257 (450)**
Somali refugees	Household sampling: We used two-stage cluster sampling using the sampling frame of the 2009 census organized by the Kenyan National Bureau of Statistics (KNBS). First, we used simple random sampling with replacement to select 40 Enumeration Areas (EAs) in Eastleigh. However, 21 EAs were deemed too insecure and dropped from the sampling frame. Following a mapping exercise of each selected EA, we randomly selected a given number households on each map.<br>Within-household sampling: Same procedure as for Somali refugees in Kakuma.	27,714 individuals (46% of Nairobi refugees)	556 (246)
Congolese refugees	Household sampling: We worked with community leaders of the Banyamulenge and Banyamasisi ethnic groups to established lists of household heads in Kasarani, Githurai, Umoja and Kayole. We focused on the two most important Congolese ethnic groups living in Nairobi: the Banyamulenge and the Banyamasisi. From the 7 lists we obtained, we used stratified simple random sampling to selected about 8% of households.<br>Within-household sampling: Same procedure as for Somali refugees in Kakuma.	16,138 individuals (27% of Nairobi refugees)	701 (204)
**Uganda Nakivale - Total (April 2018)**			**1,624 (585)**
Somali refugees	Household sampling: Using data from the 2014 census provided by the Ugandan Bureau of Statis- tics, we identified the 4 EAs in at least 30 Somali household live in Nakivale. We mapped these areas and used simple random sampling to select households.<br>Within-household sampling: Within each household, interviews were conducted with up to four adults. The household head and the main food preparer were interviewed in each household; if the number of remaining adult household members exceeded two, the remainder were chosen by random draw.	16,559 individuals (16% of settlement)	822 (290)
Congolese refugees	Household sampling: We used two-stage cluster sampling, using data from the 2014 census pro- vided by the Ugandan Bureau of Statistics. In the first stage, we randomly selected 30 enumeration areas in Nakivale sub-county using random sampling proportional to the size of the refugee pop- ulations of interest (with replacement). We then used satellite images map the selected areas and identify all households. A fixed number of households was then randomly selected in each enumeration area. Households were visited and interviewed if they were of the target nationality. Otherwise a replacement households was selected and visited.<br>Within-household sampling: Same procedure as for Somali refugees in Nakivale.	44,978 individuals (44% of settlement)	802 (295)
**Uganda Kampala - Total (April 2018)**			**932 (380)**
Somali refugees	Household sampling: We used two-stage cluster sampling, using data from the 2014 census pro- vided by the Ugandan Bureau of Statistics. In the first stage, we randomly selected 30 enumeration areas in Kampala district using random sampling proportional to the size of the refugee popu- lations of interest (with replacement). We then used satellite images and community mobilisers to map the selected areas and identify all households. A fixed number of households was then randomly selected in each enumeration area.<br>Within-household sampling: Same procedure as for Somali refugees in Nakivale.	20,545 individuals (20% of Kampala refugees)	459 (206)
Congolese refugees	Household sampling: Similar to the sampling strategy used for Somali refugees in Kampala.<br>Within-household sampling: Same procedure as for Somali refugees in Nakivale.	40,986 individuals (40% of Kampala refugees)	473 (174)
**Ethiopia Dollo Ado - Total (November-December 2018)**			**2,711 (1,152)**
Somali refugees	Household sampling: We worked in the five refugee camps spanning between Dollo Ado town and Bogol in Ethiopia (Buramino, Hilaweyn, Kobe, Melkadida, and Bokolmanyo). We used the sampling frame of UNHCR Standardised Expanded Nutrition Survey (SENS). UNHCR provided an anonymized list of the addresses of all households in the different camps. Households were randomly selected from this list using stratified simple random sampling.<br>Within-household sampling: In each selected household, we interviewed a maximum of three adults. The household head and the main food preparer were interviewed in each household; if the number of remaining adult household members exceeded one, the remainder were chosen by random draw.	218,982 individuals (100% of refugees)	2,711 (1,152)
**Ethiopia Addis Ababa - Total (September-October 2018)***			**417 (191)**
Somali refugees	Household sampling: We focused on Somali refugees living in Bole Michael. Due to the limited size of the registered population of Somali refugees in Addis Ababa, we surveyed all Somali adults living in Bole 1 and 2. The population was mobilized in 2 steps. First, all refugees in UN-HCR/ARRA database were contacted by a group of community mobilizers and asked to come to a local NGO for an interview. Many refugees could not be reached via the given phone numbers. After the list was exhausted, community mobilizers were sent to mobilize Somali refugees beyond the lists.<br>Within-household sampling: All adults were interviewed.	Census	417 (191)
**Total Refugees**			**8,303 (3,238)**

Notes: *Data collected from 693 Eritrean refugees living in Addis Ababa were excluded from the analysis because the data could not be reliably linked to ACLED data.

Pozuelo JR, et al. BMJ Ment Health 2023; 26:e300773. doi: 10.1136/bmjment-2023-300773

Table A.2 – Construction of variables

Variable	Construction
**Mental health**	
Depressive symptoms (PHQ-9 score)	The Patient Health Questionnaire-9 (PHQ-9) includes nine questions about the frequency of depressive symptoms in the two weeks preceding the survey (e.g., feeling down, trouble falling asleep, having little energy). Responses range from 0 “Not at all” to 3 “Nearly every day”. The PHQ-9 score is the sum of the nine responses, ranging from 0 to 27, with higher scores indicating greater severity of depression. The variable is standardized in regression analysis.
Prevalence of moderate to severe depression (PHQ-9 score ≥ 10)	We transform the PHQ-9 score into a binary variable using the cut-off score of 10.
Functional impairment (score)	Six questions from the WHO Disability Assessment Schedule 2.0 (WHODAS 2.0) were used to measure functional impairment in four domains of life (mobility, life activities, cognition, and participation). Respondents were asked to report on the difficulty they had achieving six different types of activity in the past 30 days, including standing for long periods, taking care of their household responsibilities, learning a new task, joining in community activities, concentrating on doing something for ten minutes, and walking a long distance. Possible answers range from 0 “No Difficulty” to 4 “Extreme difficulty”. The score is the sum of the answers to the six questions, ranging from 0 to 24, with a higher score indicating greater functional impairment. The variable is standardized in regression analysis.
**Exposure to violence**	
Exposure to violence (ACLED)	We constructed the first measure of refugees’ exposure to violence using the Armed Conflict Location & Event Data (ACLED) (Raleigh et al. 2010). ACLED data record incidents of political violence and protests reported by local, regional, or international news organisations, international organisations, NGOs, or trusted sources. We constructed an index capturing the number of violent events per 1,000 inhabitants that occurred in refugees’ districts of origin in the three years preceding their exile. We calculated the annual total of violent events in second-level administrative divisions, e.g., counties in South Sudan, districts in Somalia, and territories and cities in DRC. For data quality purposes, we only included events after the year 2000. A stylised example of a conflict event listed in ACLED reads as follows: ‘14 August 2012, the Nyatura Militia Group burned down 650 houses in a settlement in Masisi, Nord-Kivu, DRC. No fatalities were reported. Source: UNOCHA.’ ACLED data record both events and fatalities. The example shows that events with no reported fatalities might impact the population, which is why our analysis focuses on the number of events.<br>We matched ACLED data using the information on refugees’ district of origin and departure date and scaled the index using data on the population size of districts of origin based on the Gridded Population of the World (GPWv4) dataset (CIESIN 2018). The population data was extracted using AidData GeoQuery (Goodman et al. 2019). For each refugee, we used the closest population data available before their departure date. Theoretically, it is not obvious whether the number of violent events should be scaled using the population size of the district of origin, the area of the district origin, or not scaled at all. In Table A.5, we show that violence exposure best predicts depressive symptoms and functional impairment when it is scaled using the population size of refugees’ district of origin. We, therefore, used this variable in our main analysis.
Exposure to violence (self-reported)	We constructed the second measure of exposure to violence using self-reported information. Each respondent was asked whether they were ever (1) victims of violence, (2) victims of torture, or (3) witnesses of violence or torture in their country of origin or in exile. In Kenya, Uganda, and Addis Ababa, the possible answers to the three questions ranging from 0 “Not at all” to 3 “Extremely”. We construct binary variables equal to 0 for those who replied 0 “Not at all” or “A little” and 1 otherwise. In the Dollo Ado camps, possible answers were restricted to 0 “No” and 1 “Yes”. We sum the answers to the three questions to obtain a score of self-reported exposure to violence that ranges between 0 and 3.
**Socio-economic outcomes**	
Has income-generating activity (IGA)	Dummy variable equal to 1 if the respondent reported having had an activity to generate an income (self-employment, employment, business, incentive work, secondary activity) over the 30 days preceding the survey.
Monthly income (USD)	We aggregate individual incomes from work (self-employment, employment, business, incentive work, secondary activity). The final variable is expressed in 2015 USD.
Life satisfaction	The life satisfaction measure is constructed using answers to the following question: “All things considered, how satisfied are you with your life as a whole these days?”. Possible answers range from 1 “very unsatisfied” to 5 “very satisfied”.
Dietary diversity (IDDS)	The Individual Dietary Diversity Score (IDDS) is a measure of the variety of food intake, which is calculated by counting the number of twelve different food types which have been consumed at any time within the seven days preceding the survey, resulting in a score from 0 − 12 (Kennedy et al. 2011).
**Control variables**	
Age	Age of the respondent, in years.
Age squared	Age squared.
Being female	Dummy variable equal to 1 for female respondent and 0 otherwise.
Years of formal education	The number of years of education of the respondent.
Has vocational training	Dummy variable equal to 1 if the respondent completed vocational training in the past and 0 otherwise.
Has relative(s) in same site	Dummy equal to 1 if the respondent had a direct relative (brother, sister, parent, adult child) living in the same survey site.
Has relative(s) in Western countries	Dummy equal to 1 if the respondent had a direct relative (brother, sister, parent, adult child, spouse) living in a country of the Global North at the time of the survey.
Father’s years of formal education	Number of years of education of the respondent’s father, in years.
Mother’s years of formal education	Number of years of education of the respondent’s mother, in years.
Household size	Total number of household members.
Dependency ratio in household	Ratio of the number of household members to the number of adults (≥ 18y).
Sex of household head (Female =1)	Binary variable equal to 1 if the sex of the household head is female and 0 otherwise.
Years lived in current location	Number of years in exile, calculated as the difference between the year of interview and the year of arrival in the study context
Urban background (pre-exile)	Dummy variable equal to 1 for refugees who reported living in an urban environment before exile and 0 otherwise.
Living in urban context	Dummy variable equal to 1 for refugees living in Nairobi, Kampala, or Addis Ababa, and 0 otherwise
Being married/Living with partner	Dummy variable equal to 1 for respondents who are married or living with their partner, and 0 otherwise.
Has good English skills	Dummy variable based on the question “How well can you understand and speak English?.” The answers “Not at all” and “A bit” are coded as 0 and answers “Well” and “Very well” are coded as 1.
Has good local language skills	Dummy variable based on the question “How well can you understand and speak HOST LANGUAGE?”, where HOST LANGUAGE was replaced by the name of the main local language of the host population in each study context. The answers “Not at all” and “A bit” are coded as 0 and answers “Well” and “Very well” are coded as 1.

Pozuelo JR, *et al. BMJ Ment Health* 2023; 26:e300773. doi: 10.1136/bmjment-2023-300773

Table A.3 – Descriptive Statistics (Refugee Sample)

	Mean	Std.Dev.	Obs
Mental health			
Depressive symptoms (score)	6.96	5.59	8126
High depressive symptoms (binary)	0.31	0.46	8126
Functional impairment (score)	5.67	4.88	8235
Socioeconomic outcomes			
Has income-generating activity (IGA)	0.34	0.47	8298
Monthly income (USD, if IGA=1)	86.29	129.25	2661
Life satisfaction (likert scale, 1-5)	2.36	1.32	8298
Dietary Diversity (0-12)	7.22	2.54	8291
Violence exposure			
Events per 1,000 inhab. in origin district (ACLED)	0.46	0.65	7595
Self-reported measure	1.31	1.22	8054
Control variables			
Age	30.08	11.32	8298
Age squared	1033.22	842.81	8298
Being female	0.51	0.5	8298
Years of formal education	6.21	5.12	8000
Has vocational training	0.17	0.37	8298
Has relative(s) in same site	0.45	0.5	8298
Has relative(s) in Western countries	0.11	0.31	8298
Father's years of formal education	3.74	5.74	7868
Mother's years of formal education	1.92	4.12	8001
Household size (no. of people)	7.42	4.18	8278
Dependency ratio in household	1.28	1.23	8278
Female head of household	0.41	0.49	8293
Years lived in current location	5.71	4.02	8214
Urban background (pre-exile)	0.46	0.5	8239
Being married/living with partner	0.45	0.5	8298
Has good English skills	0.26	0.44	8298
Has good local language skills	0.2	0.4	8298
Events per 1,000 inhab. in origin region (ACLED)	25.1	300.29	7595

Notes: Sampling weights are accounted for when estimating the mean and the standard deviation of variables. Missing values for our main dependent and explanatory variables of interest are not imputed. For control variables included, we use dummy variable adjustment to address missing values in regression analysis.

Pozuelo JR, *et al. BMJ Ment Health* 2023; 26:e300773. doi: 10.1136/bmjment-2023-300773

Table A.4 – Descriptive Statistics by Nationality (Refugee Sample)

	Congolese			Somali			South Sudanese		
	Mean	Std.Dev.	Obs	Mean	Std.Dev.	Obs	Mean	Std.Dev.	Obs
Mental health									
Depressive symptoms (score)	10.21	5.48	2326	4.35	4.62	5337	5.91	3.95	463
High depressive symptoms (binary)	0.53	0.5	2326	0.16	0.36	5337	0.2	0.4	463
Functional impairment (score)	7.13	4.81	2392	3.74	4.02	5380	7.27	5.33	463
Socioeconomic outcomes									
Has income-generating activity (IGA)	0.5	0.5	2419	0.27	0.45	5416	0.12	0.33	463
Monthly income (USD, if IGA=1)	68.6	97.35	1264	118.39	168.48	1309	73.54	103.99	88
Life satisfaction (likert scale, 1-5)	1.6	0.82	2419	3.02	1.39	5416	2.45	1.16	463
Dietary Diversity (0-12)	7.27	2.14	2419	7.97	2.54	5409	5.07	2.22	463
Violence exposure									
Events per 1,000 inhab. in origin district (ACLED)	0.23	0.54	2182	0.83	0.68	5009	0.08	0.11	404
Self-reported measure	1.48	1.25	2371	1.05	1.21	5223	1.59	1.06	460
Control variables									
Age	30.42	11.46	2419	31.46	11.6	5416	25.49	8.78	463
Age squared	1056.57	868.36	2419	1124.29	857.19	5416	726.46	646.06	463
Being female	0.51	0.5	2419	0.55	0.5	5416	0.4	0.49	463
Years of formal education	8.09	4.75	2410	4.33	5.09	5129	6.59	4.15	461
Has vocational training	0.17	0.38	2419	0.14	0.35	5416	0.23	0.42	463
Has relative(s) in same site	0.46	0.5	2419	0.41	0.49	5416	0.56	0.5	463
Has relative(s) in Western countries	0.05	0.21	2419	0.19	0.39	5416	0.03	0.18	463
Father's years of formal education	6.69	6.07	2254	1.88	4.59	5151	1.92	4.79	463
Mother's years of formal education	3.77	5.08	2276	0.87	3.04	5262	0.46	2.13	463
Household size (no. of people)	6.85	3.03	2419	6.66	3.35	5399	10.94	6.4	460
Dependency ratio in household	1.1	0.93	2419	1.28	1.23	5399	1.74	1.71	460
Female head of household	0.28	0.45	2419	0.48	0.5	5411	0.54	0.5	463
Years lived in current location	5.45	3.94	2412	6.5	3.65	5341	4.2	4.61	461
Urban background (pre-exile)	0.41	0.49	2413	0.59	0.49	5365	0.21	0.41	461
Being married/living with partner	0.45	0.5	2419	0.48	0.5	5416	0.39	0.49	463
Has good English skills	0.15	0.36	2419	0.25	0.43	5416	0.58	0.49	463
Has good local language skills	0.26	0.44	2419	0.14	0.35	5416	0.17	0.38	463
Events per 1,000 inhab. in origin region (ACLED)	58.68	464.26	2182	1.38	1.18	5009	1.08	0.95	404

Notes: Sampling weights are accounted for when estimating the mean and the standard deviation of variables.

Table A.5 – Comparison of Different Measures of Violence Exposure

	(1)	(2)	(3)	(4)	(5)	(6)	(7)	(8)
Panel A - Dependent variable: Depressive symptoms (score, in s.d.)								
Violence exposure (ACLED events; in s.d.)	0.0208	0.0343**						
	(0.0156)	(0.0140)						
Violence exposure (ACLED events per 1000 inhabitants; in s.d.)			0.0578***	0.0579***				
			(0.0123)	(0.0114)				
Violence exposure (ACLED events per pixel; in s.d.)					0.0237	0.0339**		
					(0.0155)	(0.0144)		
Violence exposure (self-reported; in s.d.)							0.156***	0.150***
							(0.0159)	(0.0171)
N	7424	7422	7424	7422	7424	7422	7255	7253
R-sq	0.607	0.631	0.609	0.633	0.607	0.631	0.617	0.640
Panel B - Dependent variable: High depressive symptoms (binary)								
Violence exposure (ACLED events; in s.d.)	0.0205***	0.0273***						
	(0.00710)	(0.00677)						
Violence exposure (ACLED events per 1000 inhabitants; in s.d.)			0.0329***	0.0364***				
			(0.00646)	(0.00605)				
Violence exposure (ACLED events per pixel; in s.d.)					0.0184**	0.0256***		
					(0.00725)	(0.00694)		
Violence exposure (self-reported; in s.d.)							0.0543***	0.0529***
							(0.0101)	(0.0107)
N	7424	7422	7424	7422	7424	7422	7255	7253
R-sq	0.447	0.471	0.449	0.473	0.447	0.471	0.453	0.477
Panel C - Dependent variable: Functional impairment (score, in s.d.)								
Violence exposure (ACLED events; in s.d.)	0.0122	0.0274						
	(0.0173)	(0.0174)						
Violence exposure (ACLED events per 1000 inhabitants; in s.d.)			0.0591***	0.0538***				
			(0.0148)	(0.0126)				
Violence exposure (ACLED events per pixel; in s.d.)					0.0126	0.0235		
					(0.0181)	(0.0181)		
Violence exposure (self-reported; in s.d.)							0.155***	0.140***
							(0.0271)	(0.0268)
N	7532	7530	7532	7530	7532	7530	7351	7349
R-sq	0.501	0.537	0.503	0.538	0.501	0.537	0.511	0.545
Controls & enumerator FE	Yes	Yes	Yes	Yes	Yes	Yes	Yes	Yes
Enumeration area FE	No	Yes	No	Yes	No	Yes	No	Yes

Notes: This table presents the results of OLS regressions with control variables (described in Table A.3) and enumerator fixed effects. Enumeration area fixed effects are included in Columns (2), (4), (6), and (8). Different dependent variables are considered in Panel A (Depressive symptoms, as measured by the PHQ-9 score), Panel B (dummy variable equal to 1 if the PHQ-9 score ≥ 10), and Panel C (Functional impairment score). Sampling weights are included in all regressions. Cluster-robust standard errors are reported in parentheses. * p < 0.10, ** p < 0.05, *** p < 0.01

Pozuelo JR, et al. BMJ Ment Health 2023; 26:e300773. doi: 10.1136/bmjment-2023-300773

Table A.6 – Association between exposure to violence and depression, with standardized variables

	(1)	(2)	(3)	(4)
Panel A - Dependent variable: Depressive symptoms (score, in s.d.)				
Violence exposure (ACLED events per 1000 inhabitants; in s.d.)	0.0578***	0.0579***		
	(0.0123)	(0.0114)		
Violence exposure (self-reported; in s.d.)			0.156***	0.150***
			(0.0159)	(0.0171)
N	7424	7422	7255	7253
R-sq	0.609	0.633	0.617	0.640
Panel B - Dependent variable: High depressive symptoms (binary)				
Violence exposure (ACLED events per 1000 inhabitants; in s.d.)	0.0329***	0.0364***		
	(0.00646)	(0.00605)		
Violence exposure (self-reported; in s.d.)			0.0543***	0.0529***
			(0.0101)	(0.0107)
N	7424	7422	7255	7253
R-sq	0.449	0.473	0.453	0.477
Panel C - Dependent variable: Functional impairment (score, in s.d.)				
Violence exposure (ACLED events per 1000 inhabitants; in s.d.)	0.0591***	0.0538***		
	(0.0148)	(0.0126)		
Violence exposure (self-reported; in s.d.)			0.155***	0.140***
			(0.0271)	(0.0268)
N	7532	7530	7351	7349
R-sq	0.503	0.538	0.511	0.545
Controls & enumerator FE	Yes	Yes	Yes	Yes
Enumeration area FE	No	Yes	No	Yes

Notes: This table presents the results of OLS regressions with control variables (described in Table A.3) and enumerator fixed effects. Enumeration area fixed effects are included in Columns (2) and (4). Different dependent variables are considered in Panel A (Depressive symptoms, as measured by the PHQ-9 score), Panel B (dummy variable equal to 1 if the PHQ-9 score ≥ 10), and Panel C (Functional impairment score). Non-binary variables are standardized. See Tables A.9 to A.11 for full results. The results of similar regressions without standardizing variables are presented in Table A.22. Sampling weights are included in all regressions. Cluster-robust standard errors are reported in parentheses. * p < 0.10, ** p < 0.05, *** p < 0.01.

Table A.7 – Association between exposure to violence and socioeconomic outcomes, with standardized variables

	(1)	(2)	(3)	(4)
Panel A - Dependent variable: Having an IGA				
Violence exposure (ACLED events per 1000 inhabitants; in s.d.)	0.0163**	0.0124		
	(0.00677)	(0.00780)		
Violence exposure (self-reported; in s.d.)			0.0306***	0.0335***
			(0.0110)	(0.0109)
N	7588	7586	7405	7403
R-sq	0.315	0.337	0.314	0.337
Panel B - Dependent variable: Monthly income (if IGA=1, in s.d.)				
Violence exposure (ACLED events per 1000 inhabitants; in s.d.)	0.0194	0.0155		
	(0.0162)	(0.0164)		
Violence exposure (self-reported; in s.d.)			-0.123***	-0.121***
			(0.0374)	(0.0428)
N	2364	2357	2323	2316
R-sq	0.311	0.368	0.319	0.376
Panel C - Dependent variable: Monthly income (in s.d.)				
Violence exposure (ACLED events per 1000 inhabitants; in s.d.)	0.0102	0.00522		
	(0.0182)	(0.0189)		
Violence exposure (self-reported; in s.d.)			-0.0546**	-0.0556**
			(0.0232)	(0.0246)
N	7571	7569	7388	7386
R-sq	0.195	0.214	0.197	0.217
Panel D - Dependent variable: Life satisfaction (in s.d.)				
Violence exposure (ACLED events per 1000 inhabitants; in s.d.)	0.00648	0.00340		
	(0.0130)	(0.0129)		
Violence exposure (self-reported; in s.d.)			-0.121***	-0.118***
			(0.0227)	(0.0255)
N	7588	7586	7405	7403
R-sq	0.600	0.624	0.612	0.636
Panel E - Dependent variable: Dietary diversity (in s.d.)				
Violence exposure (ACLED events per 1000 inhabitants; in s.d.)	-0.0206	-0.0236**		
	(0.0132)	(0.0117)		
Violence exposure (self-reported; in s.d.)			-0.0814***	-0.0669***
			(0.0212)	(0.0191)
N	7588	7586	7405	7403
R-sq	0.584	0.647	0.592	0.653
Controls & enumerator FE	Yes	Yes	Yes	Yes
Enumeration area FE	No	Yes	No	Yes

Notes: This table presents the results of OLS regressions with control variables (described in Table A.3) and enumerator fixed effects. Enumeration area fixed effects are included in Columns (2) and (4). Different dependent variables are considered in Panel A (dummy variable equal to 1 if the respondent has a income generating activity (IGA)), Panel B (work income if the respondent has an IGA), Panel C (work income), Panel D (life satisfaction), and Panel E (dietary diversity score). Non-binary variables are standardized. See Tables A.12 to A.16 for full results. The results of similar regressions without standardizing variables are presented in Table A.23. Sampling weights are included in all regressions. Cluster-robust standard errors are reported in parentheses. * p < 0.10, ** p < 0.05, *** p < 0.01.

Table A.8 – Association between depression and socioeconomic outcomes, with standardized variables

	(1)	(2)	(3)	(4)	(5)	(6)
Panel A - Dependent variable: Having an IGA						
Depressive symptoms (score; in s.d.)	-0.00344	-0.00429				
	(0.00990)	(0.0103)				
High depressive symptoms (binary)			0.0217	0.0242		
			(0.0199)	(0.0197)		
Functional impairment (score; in s.d.)					-0.0241***	-0.0249***
					(0.00872)	(0.00871)
N	7424	7422	7424	7422	7532	7530
R-sq	0.315	0.338	0.315	0.338	0.316	0.338
Panel B - Dependent variable: Monthly income (if IGA=1, in s.d.)						
Depressive symptoms (score; in s.d.)	-0.0610**	-0.0540**				
	(0.0249)	(0.0255)				
High depressive symptoms (binary)			-0.00982	-0.0202		
			(0.0394)	(0.0405)		
Functional impairment (score; in s.d.)					-0.0737**	-0.0670**
					(0.0292)	(0.0300)
N	2299	2292	2299	2292	2349	2342
R-sq	0.322	0.377	0.320	0.376	0.312	0.369
Panel C - Dependent variable: Monthly income (in s.d.)						
Depressive symptoms (score; in s.d.)	-0.0529***	-0.0528***				
	(0.0190)	(0.0194)				
High depressive symptoms (binary)			-0.0352	-0.0363		
			(0.0355)	(0.0351)		
Functional impairment (score; in s.d.)					-0.0699***	-0.0708***
					(0.0183)	(0.0189)
N	7407	7405	7407	7405	7515	7513
R-sq	0.200	0.220	0.199	0.219	0.197	0.216
Panel D - Dependent variable: Life satisfaction (in s.d.)						
Depressive symptoms (score; in s.d.)	-0.142***	-0.157***				
	(0.0256)	(0.0230)				
High depressive symptoms (binary)			-0.187***	-0.194***		
			(0.0401)	(0.0389)		
Functional impairment (score; in s.d.)					-0.0625***	-0.0712***
					(0.0240)	(0.0233)
N	7424	7422	7424	7422	7532	7530
R-sq	0.608	0.634	0.604	0.629	0.601	0.626
Panel E - Dependent variable: Dietary diversity (in s.d.)						
Depressive symptoms (score; in s.d.)	-0.118***	-0.127***				
	(0.0190)	(0.0216)				
High depressive symptoms (binary)			-0.147***	-0.150***		
			(0.0370)	(0.0378)		
Functional impairment (score; in s.d.)					-0.0705***	-0.0749***
					(0.0183)	(0.0186)
N	7424	7422	7424	7422	7532	7530
R-sq	0.588	0.651	0.585	0.648	0.586	0.648
Controls & enumerator FE	Yes	Yes	Yes	Yes	Yes	Yes
Enumeration area FE	No	Yes	No	Yes	No	Yes

Notes: This table presents the results of OLS regressions with control variables (described in Table A.3) and enumerator fixed effects. Enumeration area fixed effects are included in Columns (2) and (4). Different dependent variables are considered in Panel A (dummy variable equal to 1 if the respondent has a income generating activity (IGA)), Panel B (work income if the respondent has an IGA), Panel C (work income), Panel D (life satisfaction), and Panel E (dietary diversity score). Non-binary variables are standardized. See Tables A.17 to A.21 for full results. The results of similar regressions without standardizing variables are presented in Table A.24. Sampling weights are included in all regressions. Cluster-robust standard errors are reported in parentheses. * p < 0.10, ** p < 0.05, *** p < 0.01.

Table A.9 – Association between exposure to violence and the depressive symptoms score, with standardized variables

	Dependent variable: Depressive symptoms (score, in s.d.)			
	(1)	(2)	(3)	(4)
Violence exposure (ACLED events per 1000 inhabitants; in s.d.)	0.0578***	0.0579***		
	(0.0123)	(0.0114)		
Violence exposure (self-reported; in s.d.)			0.156***	0.150***
			(0.0159)	(0.0171)
Age	0.0223***	0.0235***	0.0142**	0.0159***
	(0.00630)	(0.00610)	(0.00603)	(0.00591)
Age squared	-0.0000999	-0.000114	-0.0000137	-0.0000354
	(0.0000803)	(0.0000777)	(0.0000767)	(0.0000750)
Sex (Female=1)	0.0775***	0.0836***	0.0867***	0.0918***
	(0.0209)	(0.0200)	(0.0224)	(0.0211)
Education years	-0.00736**	-0.00764**	-0.00830***	-0.00894***
	(0.00306)	(0.00300)	(0.00291)	(0.00298)
Vocational training (binary)	0.0739**	0.0472	0.0556*	0.0318
	(0.0299)	(0.0295)	(0.0305)	(0.0310)
Family members in same site (binary)	-0.0643**	-0.0484*	-0.0422	-0.0273
	(0.0287)	(0.0286)	(0.0266)	(0.0264)
Family members in Global North (binary)	-0.0722**	-0.0779**	-0.0825**	-0.0865**
	(0.0339)	(0.0357)	(0.0354)	(0.0373)
Education years of father	-0.00400	-0.00370	-0.00407	-0.00380
	(0.00290)	(0.00308)	(0.00283)	(0.00307)
Education years of mother	-0.00119	-0.00166	-0.00136	-0.00166
	(0.00412)	(0.00451)	(0.00404)	(0.00432)
Household size	-0.00362	-0.000894	-0.00323	-0.000900
	(0.00522)	(0.00554)	(0.00498)	(0.00528)
Dependency ratio	0.0211*	0.0152	0.0193	0.0127
	(0.0123)	(0.0142)	(0.0118)	(0.0133)
Sex of household head (Female = 1)	0.0746***	0.0621**	0.0821***	0.0692**
	(0.0289)	(0.0316)	(0.0305)	(0.0330)
Years spent in exile	0.0124*	0.0195***	0.0145**	0.0204***
	(0.00656)	(0.00554)	(0.00660)	(0.00543)
Urban origin	-0.0577	-0.0668	-0.0298	-0.0369
	(0.0476)	(0.0452)	(0.0473)	(0.0431)
Married (binary)	0.0121	0.00190	0.00903	-0.000813
	(0.0285)	(0.0294)	(0.0277)	(0.0288)
Speaks English (binary)	-0.0701*	-0.0733*	-0.0584	-0.0593
	(0.0409)	(0.0419)	(0.0384)	(0.0391)
Speaks local language (binary)	-0.0443	-0.0626*	-0.0404	-0.0551
	(0.0354)	(0.0370)	(0.0363)	(0.0363)
N	7424	7422	7255	7253
R-sq	0.609	0.633	0.617	0.640

Notes: This table presents the results of OLS regressions with control variables and enumerator fixed effects. Enumeration area fixed effects are included in Columns (2) and (4). Sampling weights are included in all regressions. Cluster-robust standard errors are reported in parentheses. * p < 0.10, ** p < 0.05, *** p < 0.01.

Table A.10 – Association between exposure to violence and the prevalence of high depressive symptoms, with standardized

	Dependent variable: High depressive symptoms (binary)			
	(1)	(2)	(3)	(4)
Violence exposure (ACLED events per 1000 inhabitants; in s.d.)	0.0329***	0.0364***		
	(0.00646)	(0.00605)		
Violence exposure (self-reported; in s.d.)			0.0543***	0.0529***
			(0.0101)	(0.0107)
Age	0.0108***	0.0115***	0.00811**	0.00889**
	(0.00392)	(0.00395)	(0.00366)	(0.00367)
Age squared	-0.0000714	-0.0000789*	-0.0000421	-0.0000519
	(0.0000459)	(0.0000460)	(0.0000429)	(0.0000430)
Sex (Female=1)	0.0187	0.0246*	0.0223	0.0281**
	(0.0133)	(0.0128)	(0.0137)	(0.0131)
Education years	-0.000959	-0.000770	-0.00156	-0.00156
	(0.00171)	(0.00155)	(0.00172)	(0.00159)
Vocational training (binary)	0.00511	-0.00157	-0.000216	-0.00530
	(0.0201)	(0.0192)	(0.0219)	(0.0214)
Family members in same site (binary)	-0.00422	0.00152	-0.000530	0.00484
	(0.0196)	(0.0203)	(0.0176)	(0.0182)
Family members in Global North (binary)	-0.0465***	-0.0490***	-0.0488***	-0.0504***
	(0.0159)	(0.0165)	(0.0167)	(0.0174)
Education years of father	-0.00297**	-0.00280**	-0.00297**	-0.00282**
	(0.00123)	(0.00134)	(0.00123)	(0.00135)
Education years of mother	-0.00148	-0.00131	-0.00162	-0.00137
	(0.00197)	(0.00198)	(0.00196)	(0.00198)
Household size	-0.00318	-0.00180	-0.00313	-0.00200
	(0.00273)	(0.00254)	(0.00272)	(0.00254)
Dependency ratio	0.00734	0.00528	0.00783	0.00536
	(0.00582)	(0.00659)	(0.00600)	(0.00660)
Sex of household head (Female = 1)	0.0185	0.00982	0.0207	0.0116
	(0.0167)	(0.0147)	(0.0170)	(0.0151)
Years spent in exile	0.00726***	0.00925***	0.00775***	0.00924***
	(0.00270)	(0.00295)	(0.00291)	(0.00331)
Urban origin	-0.0140	-0.0161	-0.000610	-0.00145
	(0.0278)	(0.0287)	(0.0280)	(0.0285)
Married (binary)	0.0174	0.0122	0.0126	0.00696
	(0.0186)	(0.0194)	(0.0187)	(0.0198)
Speaks English (binary)	-0.0267	-0.0291	-0.0231	-0.0252
	(0.0233)	(0.0238)	(0.0228)	(0.0235)
Speaks local language (binary)	-0.0133	-0.0227	-0.00899	-0.0164
	(0.0170)	(0.0188)	(0.0171)	(0.0186)
N	7424	7422	7255	7253
R-sq	0.449	0.473	0.453	0.477

Notes: This table presents the results of OLS regressions with control variables and enumerator fixed effects. Enumeration area fixed effects are included in Columns (2) and (4). Sampling weights are included in all regressions. Cluster-robust standard errors are reported in parentheses. * p < 0.10, ** p < 0.05, *** p < 0.01.

Table A.11 – Association between exposure to violence and functional impairment, with standardized variables

	Dependent variable: Functional impairment (score, in s.d.)			
	(1)	(2)	(3)	(4)
Violence exposure (ACLED events per 1000 inhabitants; in s.d.)	0.0591***	0.0538***		
	(0.0148)	(0.0126)		
Violence exposure (self-reported; in s.d.)			0.155***	0.140***
			(0.0271)	(0.0268)
Age	-0.000711	-0.00244	-0.00845	-0.00916
	(0.00662)	(0.00654)	(0.00682)	(0.00686)
Age squared	0.000263***	0.000287***	0.000343***	0.000355***
	(0.0000836)	(0.0000840)	(0.0000857)	(0.0000868)
Sex(Female=1)	0.240***	0.245***	0.248***	0.251***
	(0.0279)	(0.0292)	(0.0300)	(0.0311)
Education years	-0.00993**	-0.00973**	-0.0103**	-0.0104**
	(0.00466)	(0.00473)	(0.00451)	(0.00470)
Vocational training (binary)	0.0335	-0.0109	0.0109	-0.0301
	(0.0607)	(0.0532)	(0.0619)	(0.0556)
Family members in same site (binary)	-0.0372	-0.0250	-0.00998	0.000452
	(0.0382)	(0.0373)	(0.0378)	(0.0372)
Family members in Global North (binary)	-0.0934**	-0.0911**	-0.0942**	-0.0896**
	(0.0393)	(0.0408)	(0.0392)	(0.0408)
Education years of father	-0.0125***	-0.0122***	-0.0128***	-0.0126***
	(0.00310)	(0.00320)	(0.00316)	(0.00326)
Education years of mother	0.00683*	0.00256	0.00558	0.00135
	(0.00357)	(0.00337)	(0.00364)	(0.00325)
Household size	-0.00276	-0.00370	-0.00178	-0.00289
	(0.00515)	(0.00501)	(0.00506)	(0.00493)
Dependency ratio	0.0328**	0.0373***	0.0301**	0.0343***
	(0.0137)	(0.0132)	(0.0129)	(0.0126)
Sex of household head (Female = 1)	0.0632*	0.0656*	0.0703*	0.0743*
	(0.0364)	(0.0391)	(0.0364)	(0.0397)
Years spent in exile	0.00564	0.0143**	0.00794	0.0153**
	(0.00853)	(0.00663)	(0.00858)	(0.00660)
Urban origin	-0.0917**	-0.104***	-0.0708*	-0.0834**
	(0.0394)	(0.0386)	(0.0388)	(0.0386)
Married (binary)	0.0579*	0.0572*	0.0621**	0.0622**
	(0.0314)	(0.0317)	(0.0290)	(0.0298)
Speaks English (binary)	-0.0147	-0.0239	0.00626	-0.000456
	(0.0516)	(0.0534)	(0.0517)	(0.0539)
Speaks local language (binary)	-0.0619	-0.0566	-0.0631	-0.0554
	(0.0426)	(0.0453)	(0.0456)	(0.0477)
N	7532	7530	7351	7349
R-sq	0.503	0.538	0.511	0.545

Notes: This table presents the results of OLS regressions with control variables and enumerator fixed effects. Enumeration area fixed effects are included in Columns (2) and (4). Sampling weights are included in all regressions. Cluster-robust standard errors are reported in parentheses. * $p < 0.10$, ** $p < 0.05$, *** $p < 0.01$.

Pozuelo JR, et al. BMJ Ment Health 2023; 26:e300773. doi: 10.1136/bmjment-2023-300773

Table A.12 – Association between exposure to violence and having an income-generating activity (IGA), with standardized variables

	Dependent variable: Having an IGA			
	(1)	(2)	(3)	(4)
Violence exposure (ACLED events per 1000 inhabitants; in s.d.)	0.0163**	0.0124		
	(0.00677)	(0.00780)		
Violence exposure (self-reported; in s.d.)			0.0306***	0.0335***
			(0.0110)	(0.0109)
Age	0.0520***	0.0538***	0.0503***	0.0521***
	(0.00476)	(0.00478)	(0.00480)	(0.00484)
Age squared	-0.000624***	-0.000647***	-0.000608***	-0.000632***
	(0.0000582)	(0.0000582)	(0.0000585)	(0.0000588)
Sex (Female=1)	-0.157***	-0.155***	-0.156***	-0.154***
	(0.0193)	(0.0196)	(0.0194)	(0.0196)
Education years	0.00251	0.00324	0.00233	0.00306
	(0.00235)	(0.00238)	(0.00248)	(0.00252)
Vocational training (binary)	0.115***	0.111***	0.113***	0.108***
	(0.0262)	(0.0277)	(0.0265)	(0.0283)
Family members in same site (binary)	-0.0242	-0.0235	-0.0207	-0.0201
	(0.0212)	(0.0207)	(0.0213)	(0.0210)
Family members in Global North (binary)	-0.0869***	-0.0919***	-0.0863***	-0.0907***
	(0.0237)	(0.0246)	(0.0242)	(0.0250)
Education years of father	-0.000374	0.000640	-0.000204	0.000839
	(0.00175)	(0.00193)	(0.00181)	(0.00199)
Education years of mother	-0.00163	-0.00217	-0.00189	-0.00247
	(0.00247)	(0.00252)	(0.00253)	(0.00258)
Household size	-0.00903***	-0.00772**	-0.00884***	-0.00754**
	(0.00289)	(0.00323)	(0.00289)	(0.00320)
Dependency ratio	0.0136**	0.00725	0.0125*	0.00572
	(0.00658)	(0.00752)	(0.00655)	(0.00748)
Sex of household head (Female = 1)	0.0303	0.0511**	0.0320	0.0531**
	(0.0222)	(0.0211)	(0.0229)	(0.0216)
Years spent in exile	0.0185***	0.0133***	0.0189***	0.0132***
	(0.00349)	(0.00372)	(0.00351)	(0.00375)
Urban origin	0.00939	0.0278	0.0144	0.0331
	(0.0225)	(0.0228)	(0.0224)	(0.0225)
Married (binary)	-0.0250	-0.0111	-0.0242	-0.00946
	(0.0155)	(0.0159)	(0.0161)	(0.0164)
Speaks English (binary)	-0.0227	-0.0132	-0.0197	-0.00875
	(0.0262)	(0.0267)	(0.0280)	(0.0288)
Speaks local language (binary)	0.0648***	0.0747***	0.0605**	0.0709***
	(0.0242)	(0.0256)	(0.0238)	(0.0252)
N	7588	7586	7405	7403
R-sq	0.315	0.337	0.314	0.337

Notes: This table presents the results of OLS regressions with control variables and enumerator fixed effects. Enumeration area fixed effects are included in Columns (2) and (4). Sampling weights are included in all regressions. Cluster-robust standard errors are reported in parentheses. * p < 0.10, ** p < 0.05, *** p < 0.01.

Table A.13 – Association between exposure to violence and income conditional on having an IGA, with standardized variables

	Dependent variable: Monthly income (if IGA=1, in s.d.)			
	(1)	(2)	(3)	(4)
Violence exposure (ACLED events per 1000 inhabitants; in s.d.)	0.0194	0.0155		
	(0.0162)	(0.0164)		
Violence exposure (self-reported; in s.d.)			-0.123***	-0.121***
			(0.0374)	(0.0428)
Age	0.0267**	0.0272**	0.0331***	0.0333***
	(0.0113)	(0.0121)	(0.0118)	(0.0127)
Age squared	-0.000256	-0.000269	-0.000320*	-0.000328*
	(0.000157)	(0.000171)	(0.000164)	(0.000178)
Sex (Female=1)	-0.145***	-0.145***	-0.138***	-0.136***
	(0.0416)	(0.0441)	(0.0401)	(0.0425)
Education years	0.0258***	0.0244***	0.0266***	0.0257***
	(0.00645)	(0.00659)	(0.00640)	(0.00664)
Vocational training (binary)	0.0721	0.104	0.0834	0.111
	(0.0805)	(0.0835)	(0.0798)	(0.0831)
Family members in same site (binary)	0.00140	0.000283	-0.0159	-0.0152
	(0.0601)	(0.0638)	(0.0593)	(0.0623)
Family members in Global North (binary)	0.147	0.107	0.147	0.105
	(0.116)	(0.122)	(0.112)	(0.118)
Education years of father	0.0128**	0.0117*	0.0127**	0.0118*
	(0.00565)	(0.00649)	(0.00569)	(0.00651)
Education years of mother	-0.0102	-0.0118	-0.00948	-0.0107
	(0.00674)	(0.00731)	(0.00700)	(0.00757)
Household size	0.00530	0.00126	0.00629	0.00201
	(0.00650)	(0.00662)	(0.00654)	(0.00657)
Dependency ratio	0.00746	0.0164	0.00312	0.00992
	(0.0215)	(0.0220)	(0.0211)	(0.0216)
Sex of household head (Female = 1)	-0.0315	-0.0314	-0.0239	-0.0162
	(0.0663)	(0.0653)	(0.0668)	(0.0664)
Years spent in exile	-0.00428	-0.00653	-0.00611	-0.00692
	(0.00881)	(0.0120)	(0.00919)	(0.0121)
Urban origin	-0.0483	-0.0114	-0.0528	-0.0229
	(0.0611)	(0.0671)	(0.0605)	(0.0656)
Married (binary)	0.216***	0.226***	0.203***	0.218***
	(0.0637)	(0.0663)	(0.0633)	(0.0673)
Speaks English (binary)	0.180**	0.181**	0.157*	0.149*
	(0.0887)	(0.0894)	(0.0894)	(0.0901)
Speaks local language (binary)	0.180**	0.198**	0.181**	0.200**
	(0.0793)	(0.0857)	(0.0802)	(0.0871)
N	2364	2357	2323	2316
R-sq	0.311	0.368	0.319	0.376

Notes: This table presents the results of OLS regressions with control variables and enumerator fixed effects. Enumeration area fixed effects are included in Columns (2) and (4). Sampling weights are included in all regressions. Cluster-robust standard errors are reported in parentheses. * p < 0.10, ** p < 0.05, *** p < 0.01.

Table A.14 – Association between exposure to violence and income, with standardized variables

	Dependent variable: Monthly income (in s.d.)			
	(1)	(2)	(3)	(4)
Violence exposure (ACLED events per 1000 inhabitants; in s.d.)	0.0102	0.00522		
	(0.0182)	(0.0189)		
Violence exposure (self-reported; in s.d.)			-0.0546**	-0.0556**
			(0.0232)	(0.0246)
Age	0.0644***	0.0646***	0.0676***	0.0678***
	(0.00847)	(0.00891)	(0.00924)	(0.00970)
Age squared	-0.000726***	-0.000731***	-0.000760***	-0.000764***
	(0.000103)	(0.000109)	(0.000111)	(0.000117)
Sex (Female=1)	-0.248***	-0.256***	-0.250***	-0.259***
	(0.0401)	(0.0410)	(0.0414)	(0.0423)
Education years	0.0263***	0.0248***	0.0270***	0.0255***
	(0.00500)	(0.00506)	(0.00521)	(0.00529)
Vocational training (binary)	0.126	0.127	0.133*	0.134*
	(0.0771)	(0.0799)	(0.0776)	(0.0805)
Family members in same site (binary)	-0.0129	-0.0162	-0.0221	-0.0261
	(0.0331)	(0.0323)	(0.0340)	(0.0333)
Family members in Global North (binary)	-0.142**	-0.171**	-0.139*	-0.168**
	(0.0710)	(0.0744)	(0.0715)	(0.0750)
Education years of father	0.00428	0.00338	0.00461	0.00354
	(0.00402)	(0.00438)	(0.00411)	(0.00448)
Education years of mother	-0.0101*	-0.0112*	-0.0105*	-0.0116*
	(0.00548)	(0.00581)	(0.00558)	(0.00591)
Household size	-0.00498	-0.00715*	-0.00478	-0.00707*
	(0.00369)	(0.00396)	(0.00370)	(0.00402)
Dependency ratio	0.0188*	0.0207*	0.0176*	0.0197*
	(0.0105)	(0.0109)	(0.0103)	(0.0107)
Sex of household head (Female = 1)	0.0107	0.0105	0.0156	0.0171
	(0.0371)	(0.0390)	(0.0382)	(0.0402)
Years spent in exile	0.0181***	0.0186**	0.0168***	0.0181**
	(0.00550)	(0.00734)	(0.00580)	(0.00752)
Urban origin	0.00245	0.0269	-0.00152	0.0214
	(0.0350)	(0.0363)	(0.0360)	(0.0372)
Married (binary)	0.171***	0.183***	0.172***	0.185***
	(0.0471)	(0.0486)	(0.0481)	(0.0498)
Speaks English (binary)	0.105*	0.116*	0.0974	0.107*
	(0.0600)	(0.0605)	(0.0606)	(0.0608)
Speaks local language (binary)	0.182***	0.174***	0.180***	0.171***
	(0.0547)	(0.0572)	(0.0557)	(0.0584)
N	7571	7569	7388	7386
R-sq	0.195	0.214	0.197	0.217

Notes: This table presents the results of OLS regressions with control variables and enumerator fixed effects. Enumeration area fixed effects are included in Columns (2) and (4). Sampling weights are included in all regressions. Cluster-robust standard errors are reported in parentheses. * p < 0.10, ** p < 0.05, *** p < 0.01.

Table A.15 – Association between exposure to violence and life satisfaction, with standardized variables

	Dependent variable: Life satisfaction (in s.d.)			
	(1)	(2)	(3)	(4)
Violence exposure (ACLED events per 1000 inhabitants; in s.d.)	0.00648<br>(0.0130)	0.00340<br>(0.0129)		
Violence exposure (self-reported; in s.d.)			-0.121***<br>(0.0227)	-0.118***<br>(0.0255)
Age	0.00130<br>(0.00479)	0.000212<br>(0.00479)	0.00789<br>(0.00547)	0.00622<br>(0.00549)
Age squared	-0.0000146<br>(0.0000572)	-0.00000475<br>(0.0000573)	-0.0000853<br>(0.0000659)	-0.0000684<br>(0.0000661)
Sex (Female=1)	-0.0238<br>(0.0160)	-0.0225<br>(0.0163)	-0.0310**<br>(0.0157)	-0.0301*<br>(0.0165)
Education years	0.00745**<br>(0.00332)	0.00538*<br>(0.00286)	0.00824**<br>(0.00333)	0.00626**<br>(0.00290)
Vocational training (binary)	-0.0531*<br>(0.0282)	-0.0551*<br>(0.0291)	-0.0462<br>(0.0301)	-0.0515*<br>(0.0312)
Family members in same site (binary)	0.0579**<br>(0.0262)	0.0425*<br>(0.0221)	0.0478*<br>(0.0270)	0.0333<br>(0.0227)
Family members in Global North (binary)	0.143***<br>(0.0302)	0.122***<br>(0.0283)	0.143***<br>(0.0306)	0.120***<br>(0.0284)
Education years of father	0.00183<br>(0.00287)	0.00227<br>(0.00332)	0.00178<br>(0.00297)	0.00216<br>(0.00351)
Education years of mother	0.00614<br>(0.00425)	0.00400<br>(0.00419)	0.00658<br>(0.00446)	0.00426<br>(0.00438)
Household size	0.00853<br>(0.00541)	0.00519<br>(0.00601)	0.00856<br>(0.00540)	0.00514<br>(0.00604)
Dependency ratio	-0.0187<br>(0.0139)	-0.0246**<br>(0.0114)	-0.0189<br>(0.0142)	-0.0240**<br>(0.0113)
Sex of household head (Female = 1)	-0.0191<br>(0.0377)	0.0155<br>(0.0239)	-0.0161<br>(0.0381)	0.0198<br>(0.0248)
Years spent in exile	0.000362<br>(0.00520)	-0.00173<br>(0.00489)	-0.00253<br>(0.00496)	-0.00312<br>(0.00464)
Urban origin	0.0251<br>(0.0540)	0.0464<br>(0.0539)	0.0232<br>(0.0506)	0.0422<br>(0.0502)
Married (binary)	-0.0345<br>(0.0338)	-0.0142<br>(0.0290)	-0.0308<br>(0.0318)	-0.00825<br>(0.0261)
Speaks English (binary)	-0.0119<br>(0.0363)	0.00569<br>(0.0369)	-0.0208<br>(0.0327)	-0.00426<br>(0.0328)
Speaks local language (binary)	0.0249<br>(0.0371)	0.0439<br>(0.0388)	0.0235<br>(0.0370)	0.0400<br>(0.0392)
N	7588	7586	7405	7403
R-sq	0.600	0.624	0.612	0.636

Notes: This table presents the results of OLS regressions with control variables and enumerator fixed effects. Enumeration area fixed effects are included in Columns (2) and (4). Sampling weights are included in all regressions. Cluster-robust standard errors are reported in parentheses. * p < 0.10, ** p < 0.05, *** p < 0.01.

Table A.16 – Association between exposure to violence and dietary diversity, with standardized variables

	Dependent variable: Dietary diversity (in s.d.)			
	(1)	(2)	(3)	(4)
Violence exposure (ACLED events per 1000 inhabitants; in s.d.)	-0.0206<br>(0.0132)	-0.0236**<br>(0.0117)		
Violence exposure (self-reported; in s.d.)			-0.0814***<br>(0.0212)	-0.0669***<br>(0.0191)
Age	0.00744<br>(0.00592)	0.00717<br>(0.00631)	0.0107*<br>(0.00637)	0.00958<br>(0.00668)
Age squared	-0.000122*<br>(0.0000734)	-0.000127<br>(0.0000777)	-0.000157**<br>(0.0000769)	-0.000153*<br>(0.0000807)
Sex (Female=1)	0.0574***<br>(0.0211)	0.0563**<br>(0.0236)	0.0525**<br>(0.0220)	0.0521**<br>(0.0245)
Education years	0.0161***<br>(0.00317)	0.0131***<br>(0.00280)	0.0171***<br>(0.00314)	0.0141***<br>(0.00277)
Vocational training (binary)	0.0369<br>(0.0298)	0.0241<br>(0.0301)	0.0489<br>(0.0308)	0.0337<br>(0.0309)
Family members in same site (binary)	0.0899**<br>(0.0446)	0.0663**<br>(0.0317)	0.0853*<br>(0.0452)	0.0636*<br>(0.0325)
Family members in Global North (binary)	0.152***<br>(0.0426)	0.120***<br>(0.0383)	0.154***<br>(0.0432)	0.121***<br>(0.0390)
Education years of father	-0.00390<br>(0.00358)	-0.00413<br>(0.00394)	-0.00389<br>(0.00361)	-0.00412<br>(0.00399)
Education years of mother	0.0204***<br>(0.00491)	0.0188***<br>(0.00454)	0.0203***<br>(0.00492)	0.0187***<br>(0.00461)
Household size	-0.00471<br>(0.00651)	-0.0135***<br>(0.00461)	-0.00508<br>(0.00644)	-0.0138***<br>(0.00446)
Dependency ratio	-0.0145<br>(0.0170)	-0.0204<br>(0.0148)	-0.0131<br>(0.0170)	-0.0191<br>(0.0149)
Sex of household head (Female = 1)	-0.0757<br>(0.0726)	-0.0123<br>(0.0386)	-0.0776<br>(0.0726)	-0.0153<br>(0.0391)
Years spent in exile	0.0186**<br>(0.00758)	0.0113*<br>(0.00681)	0.0172**<br>(0.00745)	0.0107<br>(0.00685)
Urban origin	0.120**<br>(0.0470)	0.151***<br>(0.0441)	0.111**<br>(0.0456)	0.141***<br>(0.0432)
Married (binary)	-0.00552<br>(0.0479)	0.0400<br>(0.0418)	-0.0109<br>(0.0481)	0.0355<br>(0.0416)
Speaks English (binary)	-0.0386<br>(0.0402)	-0.0158<br>(0.0396)	-0.0544<br>(0.0403)	-0.0319<br>(0.0397)
Speaks local language (binary)	0.0337<br>(0.0806)	0.0950<br>(0.0604)	0.0317<br>(0.0803)	0.0915<br>(0.0606)
N	7588	7586	7405	7403
R-sq	0.584	0.647	0.592	0.653

Notes: This table presents the results of OLS regressions with control variables and enumerator fixed effects. Enumeration area fixed effects are included in Columns (2) and (4). Sampling weights are included in all regressions. Cluster-robust standard errors are reported in parentheses. * p < 0.10, ** p < 0.05, *** p < 0.01.

Table A.17 – Association between mental health and having an IGA, with standardized variables

	Dependent variable: Having an IGA					
	(1)	(2)	(3)	(4)	(5)	(6)
Depressive symptoms (score; in s.d.)	-0.00344	-0.00429				
	(0.00990)	(0.0103)				
High depressive symptoms (binary)			0.0217	0.0242		
			(0.0199)	(0.0197)		
Functional impairment (score; in s.d.)					-0.0241***	-0.0249***
					(0.00872)	(0.00871)
Age	0.0518***	0.0538***	0.0515***	0.0534***	0.0514***	0.0531***
	(0.00480)	(0.00480)	(0.00486)	(0.00487)	(0.00476)	(0.00481)
Age squared	-0.000622***	-0.000648***	-0.000620***	-0.000645***	-0.000610***	-0.000632***
	(0.0000587)	(0.0000586)	(0.0000590)	(0.0000590)	(0.0000583)	(0.0000587)
Sex (Female=1)	-0.157***	-0.155***	-0.158***	-0.156***	-0.151***	-0.149***
	(0.0201)	(0.0203)	(0.0200)	(0.0202)	(0.0192)	(0.0194)
Education years	0.00231	0.00310	0.00236	0.00316	0.00226	0.00287
	(0.00244)	(0.00247)	(0.00243)	(0.00247)	(0.00246)	(0.00253)
Vocational training (binary)	0.115***	0.110***	0.114***	0.110***	0.116***	0.110***
	(0.0264)	(0.0280)	(0.0266)	(0.0281)	(0.0269)	(0.0283)
Family members in same site (binary)	-0.0240	-0.0219	-0.0237	-0.0218	-0.0280	-0.0267
	(0.0215)	(0.0212)	(0.0213)	(0.0210)	(0.0214)	(0.0210)
Family members in Global North (binary)	-0.0916***	-0.0962***	-0.0904***	-0.0947***	-0.0912***	-0.0954***
	(0.0245)	(0.0251)	(0.0246)	(0.0253)	(0.0243)	(0.0252)
Education years of father	-0.000286	0.000745	-0.000210	0.000827	-0.000663	0.000263
	(0.00179)	(0.00196)	(0.00179)	(0.00195)	(0.00177)	(0.00194)
Education years of mother	-0.00177	-0.00232	-0.00173	-0.00228	-0.00156	-0.00217
	(0.00256)	(0.00261)	(0.00259)	(0.00264)	(0.00251)	(0.00255)
Household size	-0.00889***	-0.00765**	-0.00882***	-0.00760**	-0.00889***	-0.00776**
	(0.00291)	(0.00329)	(0.00287)	(0.00325)	(0.00292)	(0.00327)
Dependency ratio	0.0126*	0.00599	0.0124*	0.00583	0.0131*	0.00698
	(0.00672)	(0.00765)	(0.00666)	(0.00762)	(0.00672)	(0.00753)
Sex of household head (Female = 1)	0.0275	0.0497**	0.0268	0.0491**	0.0315	0.0521**
	(0.0225)	(0.0217)	(0.0226)	(0.0216)	(0.0224)	(0.0213)
Years spent in exile	0.0184***	0.0131***	0.0182***	0.0129***	0.0180***	0.0131***
	(0.00353)	(0.00374)	(0.00351)	(0.00371)	(0.00350)	(0.00376)
Urban origin	0.0129	0.0307	0.0132	0.0311	0.0121	0.0279
	(0.0231)	(0.0228)	(0.0228)	(0.0225)	(0.0228)	(0.0227)
Married (binary)	-0.0261*	-0.0112	-0.0265*	-0.0115	-0.0235	-0.00898
	(0.0157)	(0.0160)	(0.0158)	(0.0161)	(0.0153)	(0.0157)
Speaks English (binary)	-0.0243	-0.0148	-0.0235	-0.0138	-0.0221	-0.0115
	(0.0270)	(0.0275)	(0.0270)	(0.0275)	(0.0265)	(0.0271)
Speaks local language (binary)	0.0621**	0.0730***	0.0625**	0.0738***	0.0642***	0.0745***
	(0.0250)	(0.0266)	(0.0250)	(0.0265)	(0.0242)	(0.0256)
N	7424	7422	7424	7422	7532	7530
R-sq	0.315	0.338	0.315	0.338	0.316	0.338

Notes: This table presents the results of OLS regressions with control variables and enumerator fixed effects. Enumeration area fixed effects are included in Columns (2) and (4). Sampling weights are included in all regressions. Cluster-robust standard errors are reported in parentheses. * p < 0.10, ** p < 0.05, *** p < 0.01.

Table A.18 – Association between mental health and income conditional on having an IGA, with standardized variables

	Dependent variable: Monthly income (if IGA=1, in s.d.)					
	(1)	(2)	(3)	(4)	(5)	(6)
Depressive symptoms (score; in s.d.)	-0.0610**	-0.0540**				
	(0.0249)	(0.0255)				
High depressive symptoms (binary)			-0.00982	-0.0202		
			(0.0394)	(0.0405)		
Functional impairment (score; in s.d.)					-0.0737**	-0.0670**
					(0.0292)	(0.0300)
Age	0.0310***	0.0310**	0.0299**	0.0301**	0.0277**	0.0278**
	(0.0118)	(0.0126)	(0.0117)	(0.0125)	(0.0111)	(0.0119)
Age squared	-0.000303*	-0.000314*	-0.000299*	-0.000310*	-0.000257*	-0.000267
	(0.000161)	(0.000174)	(0.000161)	(0.000175)	(0.000153)	(0.000167)
Sex (Female=1)	-0.129***	-0.137***	-0.138***	-0.145***	-0.123***	-0.125***
	(0.0423)	(0.0447)	(0.0429)	(0.0452)	(0.0408)	(0.0441)
Education years	0.0248***	0.0233***	0.0256***	0.0239***	0.0251***	0.0236***
	(0.00613)	(0.00614)	(0.00632)	(0.00632)	(0.00635)	(0.00638)
Vocational training (binary)	0.0773	0.105	0.0729	0.103	0.0741	0.101
	(0.0798)	(0.0826)	(0.0794)	(0.0823)	(0.0815)	(0.0852)
Family members in same site (binary)	-0.0106	-0.00956	-0.00354	-0.00419	-0.00468	-0.00507
	(0.0612)	(0.0652)	(0.0616)	(0.0652)	(0.0606)	(0.0648)
Family members in Global North (binary)	0.115	0.0686	0.122	0.0757	0.137	0.0960
	(0.119)	(0.127)	(0.119)	(0.126)	(0.118)	(0.125)
Education years of father	0.0122**	0.0111*	0.0128**	0.0116*	0.0124**	0.0112*
	(0.00582)	(0.00673)	(0.00583)	(0.00671)	(0.00571)	(0.00655)
Education years of mother	-0.00878	-0.0102	-0.00882	-0.0104	-0.0106	-0.0123*
	(0.00685)	(0.00753)	(0.00683)	(0.00751)	(0.00671)	(0.00726)
Household size	0.00573	0.00193	0.00601	0.00228	0.00414	0.0000141
	(0.00671)	(0.00694)	(0.00680)	(0.00698)	(0.00662)	(0.00685)
Dependency ratio	0.00592	0.0149	0.00580	0.0152	0.0131	0.0210
	(0.0220)	(0.0226)	(0.0220)	(0.0226)	(0.0227)	(0.0234)
Sex of household head (Female = 1)	-0.0191	-0.0206	-0.0248	-0.0272	-0.0206	-0.0211
	(0.0675)	(0.0677)	(0.0675)	(0.0675)	(0.0665)	(0.0663)
Years spent in exile	-0.00428	-0.00683	-0.00490	-0.00788	-0.00436	-0.00563
	(0.00915)	(0.0123)	(0.00926)	(0.0124)	(0.00924)	(0.0124)
Urban origin	-0.0555	-0.0212	-0.0551	-0.0186	-0.0411	-0.00944
	(0.0623)	(0.0682)	(0.0626)	(0.0690)	(0.0613)	(0.0662)
Married (binary)	0.217***	0.236***	0.217***	0.236***	0.222***	0.235***
	(0.0666)	(0.0703)	(0.0664)	(0.0699)	(0.0649)	(0.0673)
Speaks English (binary)	0.169*	0.178*	0.174*	0.182*	0.169*	0.172*
	(0.0940)	(0.0965)	(0.0941)	(0.0965)	(0.0890)	(0.0905)
Speaks local language (binary)	0.192**	0.209**	0.193**	0.211**	0.180**	0.197**
	(0.0840)	(0.0910)	(0.0841)	(0.0909)	(0.0801)	(0.0871)
N	2299	2292	2299	2292	2349	2342
R-sq	0.322	0.377	0.320	0.376	0.312	0.369

Notes: This table presents the results of OLS regressions with control variables and enumerator fixed effects. Enumeration area fixed effects are included in Columns (2) and (4). Sampling weights are included in all regressions. Cluster-robust standard errors are reported in parentheses. * p < 0.10, ** p < 0.05, *** p < 0.01.

Table A.19 – Association between mental health and income, with standardized variables

	Dependent variable: Monthly income (in s.d.)					
	(1)	(2)	(3)	(4)	(5)	(6)
Depressive symptoms (score; in s.d.)	-0.0529***	-0.0528***				
	(0.0190)	(0.0194)				
High depressive symptoms (binary)			-0.0352	-0.0363		
			(0.0355)	(0.0351)		
Functional impairment (score; in s.d.)					-0.0699***	-0.0708***
					(0.0183)	(0.0189)
Age	0.0666***	0.0672***	0.0658***	0.0664***	0.0639***	0.0640***
	(0.00900)	(0.00949)	(0.00889)	(0.00937)	(0.00836)	(0.00878)
Age squared	-0.000750***	-0.000760***	-0.000747***	-0.000757***	-0.000702***	-0.000705***
	(0.000108)	(0.000114)	(0.000108)	(0.000114)	(0.000100)	(0.000106)
Sex (Female=1)	-0.243***	-0.252***	-0.247***	-0.256***	-0.229***	-0.237***
	(0.0398)	(0.0409)	(0.0404)	(0.0415)	(0.0370)	(0.0379)
Education years	0.0261***	0.0246***	0.0264***	0.0250***	0.0262***	0.0246***
	(0.00497)	(0.00504)	(0.00502)	(0.00509)	(0.00494)	(0.00498)
Vocational training (binary)	0.128*	0.128	0.125	0.126	0.129	0.128
	(0.0776)	(0.0803)	(0.0778)	(0.0805)	(0.0784)	(0.0814)
Family members in same site (binary)	-0.0156	-0.0178	-0.0124	-0.0152	-0.0209	-0.0236
	(0.0332)	(0.0326)	(0.0334)	(0.0326)	(0.0336)	(0.0329)
Family members in Global North (binary)	-0.171**	-0.201***	-0.169**	-0.198**	-0.157**	-0.184**
	(0.0748)	(0.0777)	(0.0748)	(0.0776)	(0.0721)	(0.0756)
Education years of father	0.00395	0.00308	0.00405	0.00317	0.00322	0.00225
	(0.00422)	(0.00460)	(0.00421)	(0.00460)	(0.00408)	(0.00444)
Education years of mother	-0.00978*	-0.0110*	-0.00978*	-0.0110*	-0.00981*	-0.0111*
	(0.00553)	(0.00589)	(0.00556)	(0.00594)	(0.00551)	(0.00583)
Household size	-0.00501	-0.00708*	-0.00494	-0.00711*	-0.00493	-0.00729*
	(0.00375)	(0.00401)	(0.00372)	(0.00399)	(0.00368)	(0.00399)
Dependency ratio	0.0182*	0.0196*	0.0174*	0.0191*	0.0192*	0.0216*
	(0.0105)	(0.0109)	(0.0105)	(0.0110)	(0.0105)	(0.0111)
Sex of household head (Female = 1)	0.00876	0.00770	0.00522	0.00453	0.0154	0.0154
	(0.0379)	(0.0401)	(0.0378)	(0.0400)	(0.0377)	(0.0399)
Years spent in exile	0.0173***	0.0179**	0.0170***	0.0173**	0.0177***	0.0186**
	(0.00553)	(0.00723)	(0.00553)	(0.00732)	(0.00568)	(0.00735)
Urban origin	-0.000162	0.0242	0.00197	0.0267	0.00593	0.0274
	(0.0369)	(0.0378)	(0.0368)	(0.0379)	(0.0345)	(0.0354)
Married (binary)	0.171***	0.184***	0.171***	0.184***	0.175***	0.188***
	(0.0467)	(0.0483)	(0.0467)	(0.0483)	(0.0472)	(0.0486)
Speaks English (binary)	0.0946	0.106*	0.0973	0.109*	0.0988*	0.110*
	(0.0611)	(0.0616)	(0.0613)	(0.0619)	(0.0597)	(0.0601)
Speaks local language (binary)	0.183***	0.175***	0.185***	0.177***	0.179***	0.172***
	(0.0570)	(0.0600)	(0.0568)	(0.0597)	(0.0550)	(0.0577)
N	7407	7405	7407	7405	7515	7513
R-sq	0.200	0.220	0.199	0.219	0.197	0.216

Notes: This table presents the results of OLS regressions with control variables and enumerator fixed effects. Enumeration area fixed effects are included in Columns (2) and (4). Sampling weights are included in all regressions. Cluster-robust standard errors are reported in parentheses. * p < 0.10, ** p < 0.05, *** p < 0.01.

Table A.20 – Association between mental health and life satisfaction, with standardized variables

	Dependent variable: Life satisfaction (in s.d.)					
	(1)	(2)	(3)	(4)	(5)	(6)
Depressive symptoms (score; in s.d.)	-0.142***	-0.157***				
	(0.0256)	(0.0230)				
High depressive symptoms (binary)			-0.187***	-0.194***		
			(0.0401)	(0.0389)		
Functional impairment (score; in s.d.)					-0.0625***	-0.0712***
					(0.0240)	(0.0233)
Age	0.00472	0.00392	0.00358	0.00247	0.00188	0.000542
	(0.00520)	(0.00521)	(0.00523)	(0.00522)	(0.00472)	(0.00475)
Age squared	-0.0000323	-0.0000239	-0.0000316	-0.0000215	-0.00000569	0.0000100
	(0.0000603)	(0.0000603)	(0.0000611)	(0.0000611)	(0.0000574)	(0.0000581)
Sex (Female=1)	-0.0126	-0.0105	-0.0201	-0.0189	-0.0119	-0.00814
	(0.0163)	(0.0168)	(0.0165)	(0.0167)	(0.0182)	(0.0188)
Education years	0.00601*	0.00375	0.00688**	0.00481	0.00721**	0.00495*
	(0.00349)	(0.00297)	(0.00349)	(0.00297)	(0.00333)	(0.00288)
Vocational training (binary)	-0.0423	-0.0477*	-0.0517*	-0.0554*	-0.0519*	-0.0577*
	(0.0275)	(0.0284)	(0.0282)	(0.0291)	(0.0293)	(0.0303)
Family members in same site (binary)	0.0473*	0.0337	0.0557**	0.0416*	0.0545**	0.0392*
	(0.0266)	(0.0226)	(0.0270)	(0.0227)	(0.0266)	(0.0220)
Family members in Global North (binary)	0.124***	0.100***	0.125***	0.103***	0.133***	0.110***
	(0.0303)	(0.0277)	(0.0308)	(0.0283)	(0.0300)	(0.0280)
Education years of father	0.000646	0.00101	0.000654	0.00104	0.000535	0.000870
	(0.00286)	(0.00331)	(0.00289)	(0.00336)	(0.00286)	(0.00329)
Education years of mother	0.00608	0.00380	0.00597	0.00380	0.00659	0.00421
	(0.00454)	(0.00443)	(0.00455)	(0.00447)	(0.00429)	(0.00419)
Household size	0.00832	0.00529	0.00823	0.00507	0.00834	0.00474
	(0.00520)	(0.00581)	(0.00522)	(0.00586)	(0.00546)	(0.00604)
Dependency ratio	-0.0169	-0.0234**	-0.0185	-0.0247**	-0.0168	-0.0220*
	(0.0143)	(0.0110)	(0.0144)	(0.0115)	(0.0143)	(0.0117)
Sex of household head (Female = 1)	-0.0113	0.0218	-0.0186	0.0138	-0.0131	0.0222
	(0.0368)	(0.0243)	(0.0372)	(0.0251)	(0.0379)	(0.0242)
Years spent in exile	0.00227	0.00173	0.00193	0.000541	0.000598	-0.000159
	(0.00557)	(0.00462)	(0.00541)	(0.00504)	(0.00531)	(0.00468)
Urban origin	0.0187	0.0366	0.0237	0.0433	0.0260	0.0437
	(0.0554)	(0.0551)	(0.0566)	(0.0566)	(0.0550)	(0.0556)
Married (binary)	-0.0395	-0.0188	-0.0379	-0.0167	-0.0348	-0.0144
	(0.0331)	(0.0285)	(0.0335)	(0.0288)	(0.0343)	(0.0295)
Speaks English (binary)	-0.0253	-0.00963	-0.0205	-0.00397	-0.0196	-0.00247
	(0.0367)	(0.0369)	(0.0376)	(0.0385)	(0.0353)	(0.0356)
Speaks local language (binary)	0.0151	0.0313	0.0189	0.0366	0.0219	0.0405
	(0.0378)	(0.0395)	(0.0374)	(0.0396)	(0.0376)	(0.0391)
N	7424	7422	7424	7422	7532	7530
R-sq	0.608	0.634	0.604	0.629	0.601	0.626

Notes: This table presents the results of OLS regressions with control variables and enumerator fixed effects. Enumeration area fixed effects are included in Columns (2) and (4). Sampling weights are included in all regressions. Cluster-robust standard errors are reported in parentheses. * p < 0.10, ** p < 0.05, *** p < 0.01.

Table A.21 – Association between mental health and dietary diversity, with standardized variables

	Dependent variable: Dietary diversity (in s.d.)					
	(1)	(2)	(3)	(4)	(5)	(6)
Depressive symptoms (score; in s.d.)	-0.118***	-0.127***				
	(0.0190)	(0.0216)				
High depressive symptoms (binary)			-0.147***	-0.150***		
			(0.0370)	(0.0378)		
Functional impairment (score; in s.d.)					-0.0705***	-0.0749***
					(0.0183)	(0.0186)
Age	0.00957	0.00956	0.00854	0.00830	0.00781	0.00727
	(0.00597)	(0.00639)	(0.00620)	(0.00663)	(0.00600)	(0.00640)
Age squared	-0.000129*	-0.000135*	-0.000128*	-0.000132	-0.000109	-0.000109
	(0.0000734)	(0.0000776)	(0.0000764)	(0.0000811)	(0.0000744)	(0.0000786)
Sex (Female=1)	0.0656***	0.0659***	0.0592***	0.0590**	0.0726***	0.0722***
	(0.0213)	(0.0237)	(0.0213)	(0.0237)	(0.0221)	(0.0240)
Education years	0.0157***	0.0126***	0.0165***	0.0134***	0.0160***	0.0127***
	(0.00310)	(0.00272)	(0.00314)	(0.00276)	(0.00305)	(0.00274)
Vocational training (binary)	0.0470	0.0293	0.0391	0.0231	0.0382	0.0215
	(0.0301)	(0.0302)	(0.0301)	(0.0301)	(0.0304)	(0.0304)
Family members in same site (binary)	0.0831*	0.0617*	0.0901**	0.0681**	0.0871*	0.0649**
	(0.0452)	(0.0319)	(0.0455)	(0.0322)	(0.0455)	(0.0324)
Family members in Global North (binary)	0.148***	0.113***	0.150***	0.116***	0.149***	0.117***
	(0.0423)	(0.0377)	(0.0430)	(0.0384)	(0.0429)	(0.0384)
Education years of father	-0.00462	-0.00484	-0.00459	-0.00479	-0.00488	-0.00526
	(0.00362)	(0.00394)	(0.00364)	(0.00400)	(0.00363)	(0.00396)
Education years of mother	0.0205***	0.0188***	0.0205***	0.0188***	0.0209***	0.0191***
	(0.00485)	(0.00452)	(0.00501)	(0.00470)	(0.00490)	(0.00451)
Household size	-0.00537	-0.0140***	-0.00542	-0.0142***	-0.00519	-0.0141***
	(0.00625)	(0.00425)	(0.00635)	(0.00443)	(0.00660)	(0.00450)
Dependency ratio	-0.0120	-0.0181	-0.0133	-0.0191	-0.0114	-0.0164
	(0.0175)	(0.0152)	(0.0174)	(0.0148)	(0.0172)	(0.0150)
Sex of household head (Female = 1)	-0.0658	-0.00305	-0.0720	-0.00967	-0.0747	-0.0114
	(0.0728)	(0.0393)	(0.0726)	(0.0391)	(0.0725)	(0.0387)
Years spent in exile	0.0212***	0.0148**	0.0209***	0.0138*	0.0199***	0.0135**
	(0.00805)	(0.00700)	(0.00783)	(0.00706)	(0.00770)	(0.00676)
Urban origin	0.110**	0.139***	0.115**	0.145***	0.110**	0.138***
	(0.0470)	(0.0442)	(0.0475)	(0.0445)	(0.0469)	(0.0454)
Married (binary)	-0.00304	0.0436	-0.00182	0.0453	-0.00306	0.0436
	(0.0486)	(0.0425)	(0.0492)	(0.0432)	(0.0481)	(0.0417)
Speaks English (binary)	-0.0523	-0.0296	-0.0482	-0.0248	-0.0425	-0.0189
	(0.0397)	(0.0388)	(0.0401)	(0.0395)	(0.0397)	(0.0390)
Speaks local language (binary)	0.0271	0.0885	0.0304	0.0930	0.0299	0.0901
	(0.0822)	(0.0609)	(0.0814)	(0.0606)	(0.0808)	(0.0601)
N	7424	7422	7424	7422	7532	7530
R-sq	0.588	0.651	0.585	0.648	0.586	0.648

Notes: This table presents the results of OLS regressions with control variables and enumerator fixed effects. Enumeration area fixed effects are included in Columns (2) and (4). Sampling weights are included in all regressions. Cluster-robust standard errors are reported in parentheses. * p < 0.10, ** p < 0.05, *** p < 0.01.

Pozuelo JR, *et al. BMJ Ment Health* 2023; 26:e300773. doi: 10.1136/bmjment-2023-300773

Table A.22 – Association between exposure to violence and depression

	(1)	(2)	(3)	(4)
Panel A - Dependent variable: Depressive symptoms (score)				
Violence exposure (ACLED events per 1000 inhabitants)	0.510***	0.510***		
	(0.109)	(0.100)		
Violence exposure (self-reported)			0.706***	0.679***
			(0.0718)	(0.0774)
N	7424	7422	7255	7253
R-sq	0.609	0.633	0.617	0.640
Panel B - Dependent variable: High depressive symptoms (binary)				
Violence exposure (ACLED events per 1000 inhabitants)	0.0510***	0.0564***		
	(0.0100)	(0.00936)		
Violence exposure (self-reported)			0.0431***	0.0420***
			(0.00805)	(0.00850)
N	7424	7422	7255	7253
R-sq	0.449	0.473	0.453	0.477
Panel C - Dependent variable: Functional impairment (score)				
Violence exposure (ACLED events per 1000 inhabitants)	0.430***	0.392***		
	(0.107)	(0.0918)		
Violence exposure (self-reported)			0.579***	0.524***
			(0.101)	(0.0999)
N	7532	7530	7351	7349
R-sq	0.503	0.538	0.511	0.545
Controls & enumerator FE	Yes	Yes	Yes	Yes
Enumeration area FE	No	Yes	No	Yes

Notes: This table presents the results of OLS regressions with control variables (described in Table A.3) and enumerator fixed effects. Enumeration area fixed effects are included in Columns (2) and (4). Different dependent variables are considered in Panel A (Depressive symptoms, as measured by the PHQ-9 score), Panel B (dummy variable equal to 1 if the PHQ-9 score ≥ 10), and Panel C (Functional impairment score). Sampling weights are included in all regressions. Cluster-robust standard errors are reported in parentheses. * p < 0.10, ** p < 0.05, *** p < 0.01.

Table A.23 – Association between exposure to violence and socioeconomic outcomes

	(1)	(2)	(3)	(4)
Panel A - Dependent variable: Having an IGA				
Violence exposure (ACLED events per 1000 inhabitants)	0.0252**	0.0192		
	(0.0105)	(0.0121)		
Violence exposure (self-reported)			0.0243***	0.0266***
			(0.00870)	(0.00864)
N	7588	7586	7405	7403
R-sq	0.315	0.337	0.314	0.337
Panel B - Dependent variable: Monthly income in constant 2015 US$ (if IGA=1)				
Violence exposure (ACLED events per 1000 inhabitants)	3.845	3.080		
	(3.214)	(3.251)		
Violence exposure (self-reported)			-12.48***	-12.29***
			(3.808)	(4.356)
N	2364	2357	2323	2316
R-sq	0.311	0.368	0.319	0.376
Panel C - Dependent variable: Monthly income in constant 2015 US$				
Violence exposure (ACLED events per 1000 inhabitants)	1.313	0.670		
	(2.339)	(2.429)		
Violence exposure (self-reported)			-3.601**	-3.667**
			(1.531)	(1.619)
N	7571	7569	7388	7386
R-sq	0.195	0.214	0.197	0.217
Panel D - Dependent variable: Life satisfaction				
Violence exposure (ACLED events per 1000 inhabitants)	0.0140	0.00736		
	(0.0281)	(0.0279)		
Violence exposure (self-reported)			-0.135***	-0.131***
			(0.0252)	(0.0283)
N	7588	7586	7405	7403
R-sq	0.600	0.624	0.612	0.636
Panel E - Dependent variable: Dietary diversity				
Violence exposure (ACLED events per 1000 inhabitants)	-0.0790	-0.0905**		
	(0.0505)	(0.0449)		
Violence exposure (self-reported)			-0.160***	-0.132***
			(0.0417)	(0.0375)
N	7588	7586	7405	7403
R-sq	0.584	0.647	0.592	0.653
Controls & enumerator FE	Yes	Yes	Yes	Yes
Enumeration area FE	No	Yes	No	Yes

Notes: This table presents the results of OLS regressions with control variables (described in Table A.3) and enumerator fixed effects. Enumeration area fixed effects are included in Columns (2) and (4). Different dependent variables are considered in Panel A (dummy variable equal to 1 if the respondent has a income generating activity (IGA)), Panel B (work income if the respondent has an IGA), Panel C (work income), Panel D (life satisfaction), and Panel E (dietary diversity score). Sampling weights are included in all regressions. Cluster-robust standard errors are reported in parentheses. * p < 0.10, ** p < 0.05, *** p < 0.01.

Table A.24 – Association between depression and socioeconomic outcomes

	(1)	(2)	(3)	(4)	(5)	(6)
Panel A - Dependent variable: Having an IGA						
Depressive symptoms (score)	-0.000605	-0.000755				
	(0.00174)	(0.00181)				
High depressive symptoms (binary)			0.0217	0.0242		
			(0.0199)	(0.0197)		
Functional impairment (score)					-0.00512***	-0.00529***
					(0.00185)	(0.00185)
N	7424	7422	7424	7422	7532	7530
R-sq	0.315	0.338	0.315	0.338	0.316	0.338
Panel B - Dependent variable: Monthly income in constant 2015 US$ (if IGA=1)						
Depressive symptoms (score)	-1.372**	-1.215**				
	(0.561)	(0.573)				
High depressive symptoms (binary)			-1.257	-2.580		
			(5.046)	(5.191)		
Functional impairment (score)					-2.008**	-1.825**
					(0.796)	(0.816)
N	2299	2292	2299	2292	2349	2342
R-sq	0.322	0.377	0.320	0.376	0.312	0.369
Panel C - Dependent variable: Monthly income in constant 2015 US$						
Depressive symptoms (score)	-0.772***	-0.770***				
	(0.278)	(0.283)				
High depressive symptoms (binary)			-2.921	-3.011		
			(2.945)	(2.910)		
Functional impairment (score)					-1.235***	-1.250***
					(0.323)	(0.333)
N	7407	7405	7407	7405	7515	7513
R-sq	0.200	0.220	0.199	0.219	0.197	0.216
Panel D - Dependent variable: Life satisfaction						
Depressive symptoms (score)	-0.0349***	-0.0385***				
	(0.00631)	(0.00565)				
High depressive symptoms (binary)			-0.262***	-0.272***		
			(0.0561)	(0.0545)		
Functional impairment (score)					-0.0186***	-0.0212***
					(0.00713)	(0.00694)
N	7424	7422	7424	7422	7532	7530
R-sq	0.608	0.634	0.604	0.629	0.601	0.626
Panel E - Dependent variable: Dietary diversity						
Depressive symptoms (score)	-0.0513***	-0.0554***				
	(0.00829)	(0.00942)				
High depressive symptoms (binary)			-0.365***	-0.373***		
			(0.0918)	(0.0936)		
Functional impairment (score)					-0.0372***	-0.0395***
					(0.00965)	(0.00982)
N	7424	7422	7424	7422	7532	7530
R-sq	0.588	0.651	0.585	0.648	0.586	0.648
Controls & enumerator FE	Yes	Yes	Yes	Yes	Yes	Yes
Enumeration area FE	No	Yes	No	Yes	No	Yes

Notes: This table presents the results of OLS regressions with control variables (described in Table A.3) and enumerator fixed effects. Enumeration area fixed effects are included in Columns (2) and (4). Different dependent variables are con- sidered in Panel A (dummy variable equal to 1 if the respondent has a income generating activity (IGA)), Panel B (work income if the respondent has an IGA), Panel C (work income), Panel D (life satisfaction), and Panel E (dietary diversity score). Sampling weights are included in all regressions. Cluster-robust standard errors are reported in parentheses. * p < 0.10, ** p < 0.05, *** p < 0.01.

Pozuelo JR, *et al. BMJ Ment Health* 2023; 26:e300773. doi: 10.1136/bmjment-2023-300773

Table A.25 – Association between self-reported exposure to violence and depression, with standardized variables

	(1)	(2)	(3)	(4)	(5)	(6)	(7)	(8)	(9)	(10)
Panel A - Dependent variable: Depressive symptoms (score, in s.d.)										
Violence exposure (self-reported; in s.d.)	0.156***	0.150***								
	(0.0159)	(0.0171)								
Direct exposure to violence			0.237***	0.234***					0.140***	0.147***
			(0.0301)	(0.0308)					(0.0332)	(0.0353)
Direct exposure to torture					0.257***	0.238***			0.177***	0.157***
					(0.0313)	(0.0333)			(0.0343)	(0.0356)
Witnessed violence or torture							0.162***	0.154***	0.0529**	0.0490*
							(0.0280)	(0.0296)	(0.0250)	(0.0264)
N	7255	7253	7310	7308	7305	7303	7272	7270	7255	7253
R-sq	0.617	0.640	0.613	0.637	0.614	0.637	0.609	0.632	0.618	0.641
Panel B - Dependent variable: High depressive symptoms (binary)										
Violence exposure (self-reported; in s.d.)	0.0543***	0.0529***								
	(0.0101)	(0.0107)								
Direct exposure to violence			0.0946***	0.0955***					0.0607**	0.0646**
			(0.0202)	(0.0202)					(0.0259)	(0.0259)
Direct exposure to torture					0.102***	0.0963***			0.0745***	0.0671**
					(0.0232)	(0.0248)			(0.0269)	(0.0281)
Witnessed violence or torture							0.0389**	0.0366**	-0.00823	-0.00939
							(0.0158)	(0.0175)	(0.0172)	(0.0192)
N	7255	7253	7310	7308	7305	7303	7272	7270	7255	7253
R-sq	0.453	0.477	0.451	0.474	0.451	0.474	0.447	0.472	0.455	0.479
Panel C - Dependent variable: Functional impairment (score, in s.d.)										
Violence exposure (self-reported; in s.d.)	0.155***	0.140***								
	(0.0271)	(0.0268)								
Direct exposure to violence			0.227***	0.213***					0.144**	0.147**
			(0.0484)	(0.0477)					(0.0638)	(0.0651)
Direct exposure to torture					0.205***	0.172***			0.111	0.0848
					(0.0578)	(0.0573)			(0.0692)	(0.0693)
Witnessed violence or torture							0.202***	0.182***	0.112***	0.100**
							(0.0353)	(0.0365)	(0.0409)	(0.0410)
N	7351	7349	7416	7414	7410	7408	7368	7366	7351	7349
R-sq	0.511	0.545	0.508	0.543	0.506	0.541	0.506	0.541	0.511	0.545
Controls & enumerator FE	Yes	Yes	Yes	Yes	Yes	Yes	Yes	Yes	Yes	Yes
Enumeration area FE	No	Yes	No	Yes	No	Yes	No	Yes	No	Yes

Notes: This table presents the results of OLS regressions with control variables (described in Table A.3) and enumerator fixed effects. Enumeration area fixed effects are included in Columns (2), (4), (6), (8), and (10). Different dependent variables are considered in Panel A (Depressive symptoms, as measured by the PHQ-9 score), Panel B (dummy variable equal to 1 if the PHQ-9 score ≥ 10), and Panel C (Functional impairment score). Non-binary variables are standardized. Sampling weights are included in all regressions. Cluster-robust standard errors are reported in parentheses. * $p < 0.10$, ** $p < 0.05$, *** $p < 0.01$.

Table A.26 – Association between self-reported exposure to violence and socioeconomic outcomes, with standardized variables

	(1)	(2)	(3)	(4)	(5)	(6)	(7)	(8)	(9)	(10)
Panel A - Dependent variable: Having an IGA										
Violence exposure (self-reported; in s.d.)	0.0306***	0.0335***								
	(0.0110)	(0.0109)								
Direct exposure to violence			0.0140	0.00775					-0.0275	-0.0421*
			(0.0201)	(0.0207)					(0.0235)	(0.0237)
Direct exposure to torture					0.0539***	0.0649***			0.0511**	0.0678***
					(0.0186)	(0.0173)			(0.0200)	(0.0187)
Witnessed violence or torture							0.0579***	0.0626***	0.0526**	0.0590**
							(0.0207)	(0.0208)	(0.0230)	(0.0233)
N	7405	7403	7471	7469	7465	7463	7422	7420	7405	7403
R-sq	0.314	0.337	0.313	0.335	0.315	0.338	0.315	0.337	0.316	0.340
Panel B - Dependent variable: Monthly income (if IGA=1, in s.d.)										
Violence exposure (self-reported; in s.d.)	-0.123***	-0.121***								
	(0.0374)	(0.0428)								
Direct exposure to violence			-0.163***	-0.163**					-0.0827	-0.0948
			(0.0616)	(0.0690)					(0.0602)	(0.0648)
Direct exposure to torture					-0.153**	-0.133**			-0.0850	-0.0632
					(0.0594)	(0.0592)			(0.0570)	(0.0553)
Witnessed violence or torture							-0.185***	-0.189***	-0.127**	-0.135**
							(0.0586)	(0.0711)	(0.0497)	(0.0599)
N	2323	2316	2348	2341	2348	2341	2329	2322	2323	2316
R-sq	0.319	0.376	0.315	0.373	0.315	0.372	0.317	0.374	0.319	0.376
Panel C - Dependent variable: Monthly income (in s.d.)										
Violence exposure (self-reported; in s.d.)	-0.0546**	-0.0556**								
	(0.0232)	(0.0246)								
Direct exposure to violence			-0.0925***	-0.0984***					-0.0657**	-0.0758**
			(0.0323)	(0.0337)					(0.0321)	(0.0331)
Direct exposure to torture					-0.0658	-0.0545			-0.0241	-0.00691
					(0.0404)	(0.0404)			(0.0403)	(0.0397)
Witnessed violence or torture							-0.0717*	-0.0798*	-0.0393	-0.0488
							(0.0407)	(0.0444)	(0.0378)	(0.0411)
N	7388	7386	7454	7452	7448	7446	7405	7403	7388	7386
R-sq	0.197	0.217	0.195	0.214	0.197	0.216	0.196	0.215	0.197	0.217
Panel D - Dependent variable: Life satisfaction (in s.d.)										
Violence exposure (self-reported; in s.d.)	-0.121***	-0.118***								
	(0.0227)	(0.0255)								
Direct exposure to violence			-0.210***	-0.211***					-0.150***	-0.156***
			(0.0556)	(0.0594)					(0.0533)	(0.0537)
Direct exposure to torture					-0.167***	-0.165***			-0.0783***	-0.0803***
					(0.0382)	(0.0412)			(0.0257)	(0.0261)
Witnessed violence or torture							-0.139***	-0.121***	-0.0572*	-0.0379
							(0.0281)	(0.0289)	(0.0315)	(0.0281)
N	7405	7403	7471	7469	7465	7463	7422	7420	7405	7403
R-sq	0.612	0.636	0.609	0.633	0.606	0.630	0.608	0.632	0.613	0.637
Panel E - Dependent variable: Dietary diversity (in s.d.)										
Violence exposure (self-reported; in s.d.)	-0.0814***	-0.0669***								
	(0.0212)	(0.0191)								
Direct exposure to violence			-0.113***	-0.121***					-0.0620*	-0.0979***
			(0.0325)	(0.0280)					(0.0372)	(0.0281)
Direct exposure to torture					-0.129***	-0.118***			-0.0892**	-0.0838**
					(0.0347)	(0.0310)			(0.0371)	(0.0340)
Witnessed violence or torture							-0.0936**	-0.0348	-0.0420	0.0290
							(0.0364)	(0.0302)	(0.0393)	(0.0313)
N	7405	7403	7471	7469	7465	7463	7422	7420	7405	7403
R-sq	0.592	0.653	0.590	0.653	0.590	0.653	0.590	0.651	0.592	0.654
Controls & enumerator FE	Yes	Yes	Yes	Yes	Yes	Yes	Yes	Yes	Yes	Yes
Enumeration area FE	No	Yes	No	Yes	No	Yes	No	Yes	No	Yes

Notes: This table presents the results of OLS regressions with control variables (described in Table A.3) and enumerator fixed effects. Enumeration area fixed effects are included in Columns (2), (4), (6), (8), and (10). Different dependent variables are considered in Panel A (dummy variable equal to 1 if the respondent has a income generating activity (IGA)), Panel B (work income if the respondent has an IGA), Panel C (work income), Panel D (life satisfaction), and Panel E (dietary diversity score). Non-binary variables are standardized. Sampling weights are included in all regressions. Cluster-robust standard errors are reported in parentheses. * p < 0.10, ** p < 0.05, *** p < 0.01.

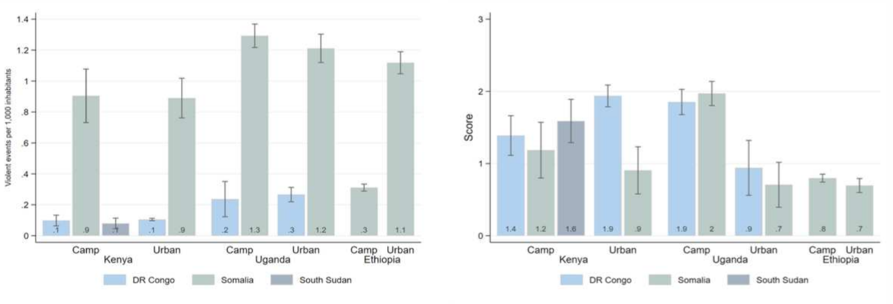

(a) Exposure to the violent events recorded in the ACLED database

(b) Self-reported exposure to violence or torture

Figure A.1 – Exposure to violence

Note: For each refugee strata, Figure (a) presents the average number of violent events that the ACLED project recorded in refugees' districts of origin during the three years prior to their departure. The measure is expressed per 1,000 inhabitants. Data on population size come from the AIDDATA project. For each refugee strata, Figure (b) presents the average of an index that aggregates respondents' answers to three questions asking whether respondents were ever (1) victim of violence, (2) victim of torture, or (3) witnesses of violence or torture in their country of origin or in exile, with answers coded as 0 for "No" and 1 for "Yes". The index is the sum the answers and ranges between 0 and 3. Data come from the Refugee Economies Dataset.

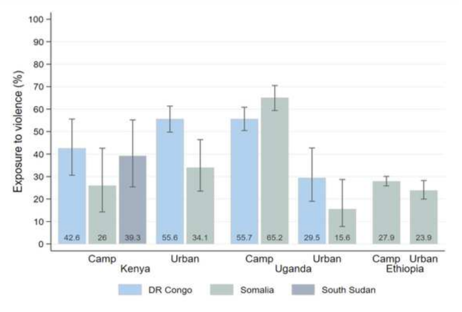

(a) Direct exposure to violence

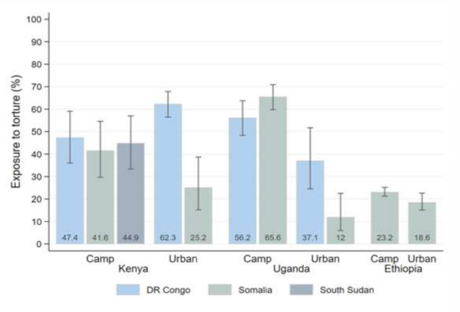

(b) Direct exposure to torture

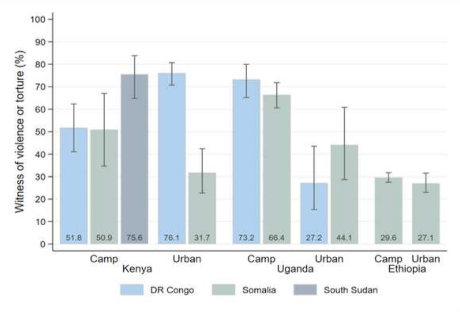

(c) Witnessed violence or torture

Figure A.2 – Components of self-reported exposure to violence

Note: For each refugee strata, the figures presents the proportion of respondents who self-reported being exposed or witnessing violence or torture in their country of origin or in exile. Data come from the Refugee Economies Dataset.

STROBE Statement—Checklist of items that should be included in reports of ***cross-sectional studies***

	**Item No**	**Recommendation**	**Page No**
**Title and abstract**	1	(*a*) Indicate the study's design with a commonly used term in the title or the abstract	1
		(*b*) Provide in the abstract an informative and balanced summary of what was done and what was found	2
**Introduction**			
Background/rationale	2	Explain the scientific background and rationale for the investigation being reported	2-4
Objectives	3	State specific objectives, including any prespecified hypotheses	2-4
**Methods**			
Study design	4	Present key elements of study design early in the paper	4-5
Setting	5	Describe the setting, locations, and relevant dates, including periods of recruitment, exposure, follow-up, and data collection	4-5
Participants	6	(*a*) Give the eligibility criteria, and the sources and methods of selection of participants	4-5
Variables	7	Clearly define all outcomes, exposures, predictors, potential confounders, and effect modifiers. Give diagnostic criteria, if applicable	5-6, Supplement
Data sources/ measurement	8*	For each variable of interest, give sources of data and details of methods of assessment (measurement). Describe comparability of assessment methods if there is more than one group	5-6, Supplement
Bias	9	Describe any efforts to address potential sources of bias	5-6, Supplement
Study size	10	Explain how the study size was arrived at	4-5 Supplement
Quantitative variables	11	Explain how quantitative variables were handled in the analyses. If applicable, describe which groupings were chosen and why	4-5 Supplement
Statistical methods	12	(*a*) Describe all statistical methods, including those used to control for confounding	6-7
		(*b*) Describe any methods used to examine subgroups and interactions	6-7
		(*c*) Explain how missing data were addressed	Supplement
		(*d*) If applicable, describe analytical methods taking account of sampling strategy	6-7 Supplement
		(*e*) Describe any sensitivity analyses	6-7 Supplement
**Results**			
Participants	13*	(a) Report numbers of individuals at each stage of study—eg numbers potentially eligible, examined for eligibility, confirmed eligible, included in the study, completing follow-up, and analysed	4, Supplement
		(b) Give reasons for non-participation at each stage	Not relevant
		(c) Consider use of a flow diagram	Not relevant
Descriptive data	14*	(a) Give characteristics of study participants (eg demographic, clinical, social) and information on exposures and potential	7-8, Supplement

		confounders	
		(b) Indicate number of participants with missing data for each variable of interest	Supplement
Outcome data	15*	Report numbers of outcome events or summary measures	7-8, Supplement
Main results	16	(*a*) Give unadjusted estimates and, if applicable, confounder-adjusted estimates and their precision (eg, 95% confidence interval). Make clear which confounders were adjusted for and why they were included	7-8, Supplement
		(*b*) Report category boundaries when continuous variables were categorized	7-8, Supplement
		(*c*) If relevant, consider translating estimates of relative risk into absolute risk for a meaningful time period	Not relevant
Other analyses	17	Report other analyses done—eg analyses of subgroups and interactions, and sensitivity analyses	7-8, Supplement
**Discussion**			
Key results	18	Summarise key results with reference to study objectives	8-9
Limitations	19	Discuss limitations of the study, taking into account sources of potential bias or imprecision. Discuss both direction and magnitude of any potential bias	8
Interpretation	20	Give a cautious overall interpretation of results considering objectives, limitations, multiplicity of analyses, results from similar studies, and other relevant evidence	8-9
Generalisability	21	Discuss the generalisability (external validity) of the study results	9
**Other information**			
Funding	22	Give the source of funding and the role of the funders for the present study and, if applicable, for the original study on which the present article is based	9

*Give information separately for exposed and unexposed groups.

Note: An Explanation and Elaboration article discusses each checklist item and gives methodological background and published examples of transparent reporting. The STROBE checklist is best used in conjunction with this article (freely available on the Web sites of PLoS Medicine at http://www.plosmedicine.org/, Annals of Internal Medicine at http://www.annals.org/, and Epidemiology at http://www.epidem.com/). Information on the STROBE Initiative is available at www.strobe-statement.org.
